# Overexpression of Global Regulator SCrp Leads to the Discovery of New Angucyclines in *Streptomyces* sp. XS-16

**DOI:** 10.3390/md21040240

**Published:** 2023-04-14

**Authors:** Xiao Xu, Falei Zhang, Luning Zhou, Yimin Chang, Qian Che, Tianjiao Zhu, Dehai Li, Guojian Zhang

**Affiliations:** 1 Key Laboratory of Marine Drugs, Chinese Ministry of Education, School of Medicine and Pharmacy, Ocean University of China, Qingdao 266003, China; 2 Laboratory for Marine Drugs and Bioproducts, Pilot National Laboratory for Marine Science and Technology, Qingdao 266237, China; 3 Marine Biomedical Research Institute of Qingdao, Qingdao 266101, China

**Keywords:** overexpression, global regulator, SCrp, angucyclines, *Streptomyces* sp.

## Abstract

Six angucyclines including three unreported compounds (**1**–**3**) were isolated from *Streptomyces* sp. XS-16 by overexpressing the native global regulator of SCrp (cyclic AMP receptor). The structures were characterized based on nuclear magnetic resonance (NMR) and spectrometry analysis and assisted by electronic circular dichroism (ECD) calculations. All compounds were tested for their antitumor and antimicrobial activities, and compound **1** showed different inhibitory activities against various tumor cell lines with IC_50_ values ranging from 0.32 to 5.33 μM.

## 1. Introduction

*Streptomyces* have been demonstrated to be the producers of many clinically important antibiotics, such as tetracyclines, glycopeptides, macrolides, and polypeptides [1,2]; however, genome sequencing in recent years has shown that the metabolic potentials of *Streptomyces* are far from being fully exploited with a large group of gene clusters remaining cryptic or poorly expressed when strains are cultivated in laboratory [3,4,5,6]. Therefore, various methods such as OSMAC (one strain many compounds) [7], ribosome engineering [8], heterologous expression [9,10], coculture [11], promoter engineering [12], and metabolic regulation [13,14,15] have been developed to awaken or upregulate their expression as well as for the identification of molecules encoded by these gene clusters.

The cyclic AMP-receptor protein (Crp) is a global transcription regulator broadly distributed in a variety of bacteria and regulates multiple biological activities such as glucose starvation, cell differentiation, and primary metabolism [16]. Crp plays a key role in spore germination and colony development of *Streptomyces*, and it can regulate primary metabolism and enhance the precursor flux to secondary metabolite biosynthesis [17,18,19,20]. Thus, Crp was identified as the overall regulator of primary metabolism that stimulates the production of secondary metabolites. Studies have shown that overexpression of Crp in a group of *Streptomyces* leads to enhanced antibiotic biosynthesis and the production of new metabolites such as high production of actinorhodin (an aromatic polyketide antibiotic), undecylprodigiosin (a tripyrrolic pigment such as prodigiosin with antimalarial, antiulcer, and apoptotic activities), and a calcium-dependent antibiotic in *S. coelicolor*, indicating that the regulation of Crp on secondary metabolism is widely conserved in *Streptomyces* [20]. In addition, overexpression of Crp has been shown to successfully stimulate the production of monensin (a polyether ionophore antibiotic widely used as a coccidiostat and a growth-promoting agent in agricultural industry) in *S. cinnamonensis* [21] and daptomycin (a novel cyclic lipopeptide antibiotic that is effective against Gram-positive bacteria) in *S. roseosporus* [22].

During our ongoing work in searching for bioactive compounds from marine-derived microbes, an actinomycete strain *Streptomyces* sp. XS-16 was obtained from marine sediment samples, collected from the sea around Naozhou Island in Zhanjiang, Guangdong Province. Genomic sequencing and analysis revealed a 7.54 Mb genome for the strain XS-16; however, we only obtained two previously described compounds named flazaine [23] and 1-(2-hydroxyphenyl)-2-phenylethanone [24] under laboratory cultivation. Thus, in order to tap into the metabolic potential and obtain diversified secondary metabolites, we overexpressed SCrp, the native global regulatory factor of Crp discovered by genome mining from XS-16. As a result, the metabolic profile was diversified and six typical angucycline derivatives, including three undescribed ones, were isolated from the mutant strain *Streptomyces* sp. XS-16-SCrp. Among the isolated compounds, **1**, contains an oxygen-containing ternary ring system, and showed different inhibitory activities against five cancer cell lines with IC_50_ values ranging from 0.32 to 5.33 µM. Here, we report the details of metabolic profile modulation by overexpression of SCrp, the isolation and characterization of new agucyclines, as well as bioactivity evaluation of the isolated compounds.

## 2. Results and Discussion

**Figure 1:**
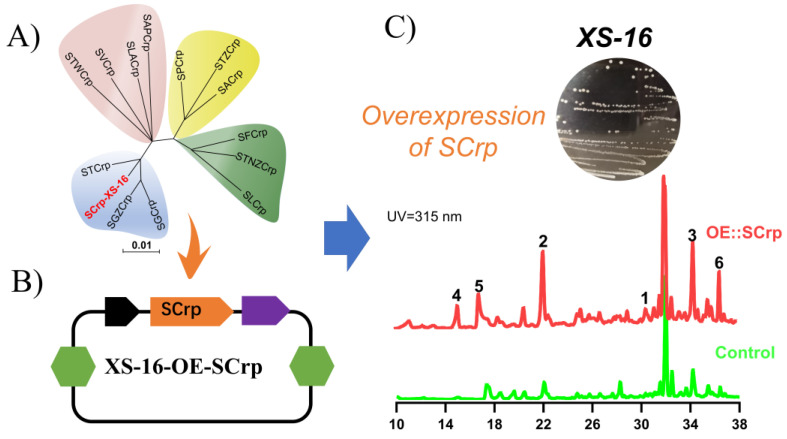
(**A**) Phylogenetic analysis of SCrp. (**B**) The plasmid of XS-16-OE-SCrp (**C**) HPLC analysis of the extracts from the control strain and OE::SCrp strain of *Streptomyces* sp. XS-16. The GenBank number of SCrp homologues in the NCBI database: SGCrp WP 257623897 *S. griseus*; SGZCrp P 257623897 *S. griseus*; STCrp WP 202418726 *Streptomyces* sp.; STWCrp WP 202467577 *Streptomyces* sp.; SLCrp WP 217502119 *S. lunaelactis*; STZCrp WP 202472685 *Streptomyces* sp.; SPCrp WP 236064049 *S. poriferorum*; SVCrp ARS88281 *S. virginiae*; SACrp WP 010986016 *S. avermitilis*; STNZCrp WP 077966648 *S. tsukubensis*; SAPCrp WP 156725626 *S. apocynin*; SFCrp WP 156693126 *S. ficellus*; SLACrp QUQ55778 *S. lavendulae*.

The Crp gene analogue, SCrp, was identified via Localblast, using a Crp gene (*Streptomyces lividans* TK24, AIJ15031.1) as a query. The total size of the gene is 675 bp, which encodes a polypeptide of 224 amino acids. The BLAST analysis by NCBI indicated that the SCrp protein had 99% sequence identity to the protein of SGCrp (*S. griseus* WP_257623897.1), SGZCrp (*S. griseus* WP 257623897), STCrp (*Streptomyces* sp. WP 202418726). Phylogenetic analysis revealed that SCrp is mostly related to STCrp (Figure S2). Sequence analysis via InterProScan showed that the SCrp protein possessed two domains: cyclic nucleotide monophosphate binding domain and Crp-type HTH domain profile, which are consistent with the putative mechanism of Crp genes [25,26]. The SCrp in *Streptomyces* sp. XS-16 was amplified by specific primers (Table S1) and ligated into the lined vector pSET152 (cuted by the restriction site BamHI) using the ClonExpress Ultra One Step Cloning Kit C115 (Vazyme). The recombinant vector was transferred to *Streptomyces* sp. XS-16 to generate the OE::SCrp mutants (the mutant of *Streptomyces* sp. XS-16 harboring vacant pSET152 was also generated as a control). Following a fermentation in M1 media with shaking at 28 °C for 8 days, high-performance liquid chromatography (HPLC) analysis also showed a series of new peaks present in the extract of the OE::SCrp mutant compared with that of the control strain (Figure 1), indicating changes in secondary metabolite production.

**Figure 2:**
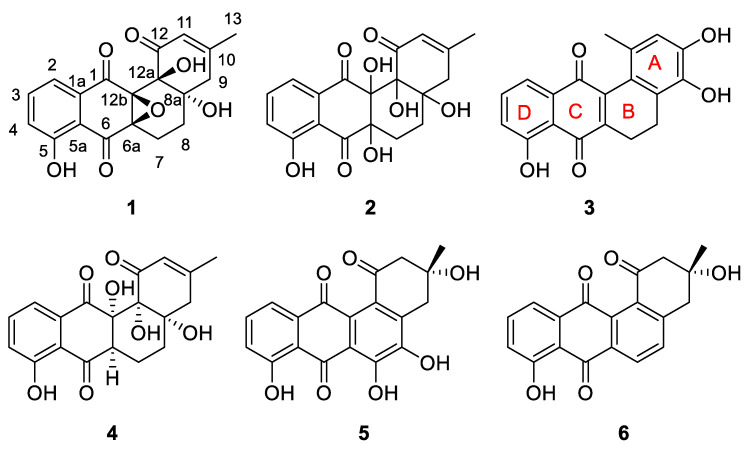
Chemical structures of **1**−**6**.

To explore the structures for the activation products, the overexpression strain, *Streptomyces* sp. OE::SCrp, was cultured on a larger scale (30 L). Guided by HPLC data, the EtOAc extract (3.2 g) of the fermentation was fractionated by octadecyl silane chemically bonded silica (ODS), medium pressure liquid chromatography (MPLC) and then HPLC to yield three undescribed compounds: **1** (3.0 mg), **2** (7.0 mg) and **3** (7.0 mg). The previously described compounds **4**–**6** were obtained and identified by comparison of their 1D NMR and MS data with those reported in the literature (Figure 2).

**Figure 3:**
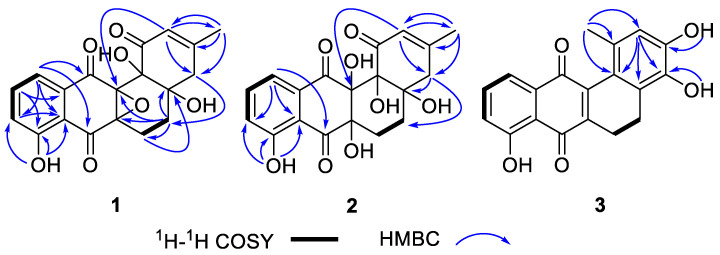
^1^H−^1^H COSY and key HMBC correlations of **1**–**3**.

Compound **1** was obtained as a pale yellowish powder. Its molecular formula C_19_H_16_O_7_ was determined on the basis of the HR-ESIMS *m*/*z* 355.0824 [M − H]^−^ (calcd. for C_19_H_15_O_7_^−^, 355.0823), corresponding to twelve degrees of unsaturation. The infrared (IR) spectrum of **1** displayed intense absorption bands at 3392 and 1655 cm^−1^, suggesting the presence of hydroxyl and carbonyl groups, respectively. The ^1^H NMR spectrum of **1** displayed signals for methyl (*δ*_H_ 1.86, s), methylenes (*δ*_H_ 2.80, 2.26; *δ*_H_ 2.70, 2.26; *δ*_H_ 1.82, 1.49), vinyl methine (*δ*_H_ 5.91, s), aromatic methines (*δ*_H_ 7.71, dd, *J* = 8.4, 7.6 Hz; *δ*_H_ 7.41, dd, *J* = 7.6, 1.0 Hz; *δ*_H_ 7.34, dd, *J* = 8.4, 1.0 Hz), and a signal for one exchangeable proton (*δ*_H_ 11.16, s) (Table 1). The ^13^C NMR spectrum, in combination with the HSQC spectrum, indicates the presence of 1 methyl (*δ*_C_ 23.4), 3 sp3 methylenes (*δ*_C_ 17.4, 27.5, and 41.5), 4 protonated sp2 carbons (*δ*_C_ 118.8, 121.7, 124.3, and 136.7), and 11 quaternary carbons including 3 non-protonated sp3 carbons (*δ*_C_ 63.6, 72.8, and 75.2), 5 non-protonated sp2 carbons (*δ*_C_ 109.5, 114.3, 131.9, 159.2, and 159.8), and 3carbonyls (*δ*_C_ 189.8, 194.0, and 195.5) (Table 1). These data show that **1** has a tetracyclic benz[*a*]anthracene system and belongs to the angucycline class [27]. Subsequently, the planar structure of **1** was determined by detailed analysis of its 2D NMR data. Firstly, the ^1^H-^1^H COSY correlations from H-2 (*δ*_H_ 7.41, d, *J* = 7.6 Hz)/H-3 (*δ*_H_ 7.71, t, *J* = 8.0 Hz)/H-4 (*δ*_H_ 7.34, dd, *J* = 8.4, 1.0 Hz) suggested the presence of the 1,2,3-trisubstituted benzene ring (ring D) (Figure 3). The HMBC correlations from 5-OH (*δ*_H_ 11.16, s) to C-4 (*δ*_C_ 124.3, d), C-5 (*δ*_C_ 159.8, s), and C-5a (*δ*_C_ 114.3, s) revealed that the phenolic OH was on C-5. The A ring was determined using the HMBC correlations from Me-13 to C-9 (*δ*_C_ 41.5, t), C-10 (*δ*_C_ 159.2, s), and C-11 (*δ*_C_ 121.7, d) in addition to the HMBC correlations from H-11 (*δ*_H_ 5.91, s) to C-9, C-12b (*δ*_C_ 75.2, s), and C-13 (*δ*_C_ 23.4, q). The B ring was determined using the ^1^H-^1^H COSY correlations from H-7 to H-8 in addition to the HMBC correlations from H-7β (*δ*_H_ 2.27, m) to C-8a (*δ*_C_ 63.6, s), and from H-8β (*δ*_H_ 1.49, m) to C-6a (*δ*_C_ 72.8, s), C-7 (*δ*_C_ 17.4, t), C-8a, and C-12b. The HMBC correlations from H-11 and H-8β to C-12b, from H-7β to C-8a, and from H-9β (*δ*_H_ 2.24, m) to C-8 suggested the connectivity of the A and B rings. The quinone-like C ring was determined using the HMBC correlations from H-2 to C-1 (*δ*_C_ 189.8, s) and C-6 (*δ*_C_ 194.0, s). Four carbon-carbon double bonds (*δ*_C_ 131.9, 118.8, 136.7, 124.3, 159.8, 114.3, 159.2 and 121.7), three carbonyl groups (*δ*_C_ 189.8, 194.0 and 195.5), and tetracyclic skeleton, accounting for eleven degrees of unsaturation, then another degree of unsaturation should be the existence of an epoxide between C-6a (*δ*_C_ 72.8, s) and C-12b. Therefore, the planar structure of **1** was determined (Figure 2). 

**Figure 4:**
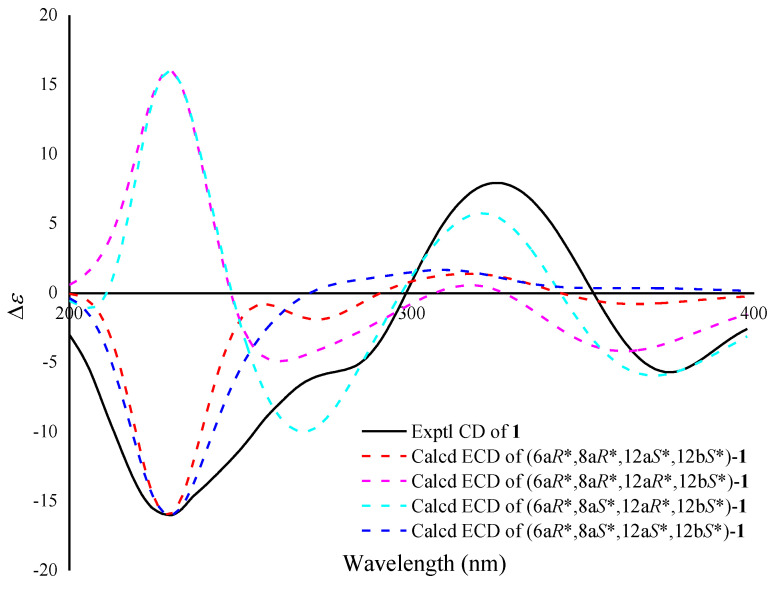
Calculated and experimental ECD spectra of **1**.

However, as the relative configuration of the epoxide and the two hydroxyl groups could not be defined by the ROESY data, we resorted to ECD analysis and computational chemistry method. The ECD spectra of four possible structures (6aR*,8aR*,12aS*,12b*S*)-**1**, (6aR*,8aR*,12aR*,12b*S*)-**1**, (6aR*,8aS*,12aR*,12b*S*)-**1** and (6aR*,8aS*,12aS*,12bS*)-**1** were calculated at the B3LYP/6-31+G(d,p)/B3LYP/6-31G(d) level and was compared with the experimental ECD spectrum. As shown in Figure 4, the calculated ECD spectrum of (6aR*,8aR*,12aS*,12b*S*)-**1** and (6aR*,8aS*,12aS*,12bS*)-**1** matched well with the experimental one. Then, the theoretical NMR calculations and DP4+ probability analyses were employed to determine its final structure. The ^13^C NMR chemical shifts of (6aR*,8aR*,12aS*,12b*S*)-**1** and (6aR*,8aS*,12aS*,12bS*)-**1** were calculated at the B3LYP/6-311+G(d,p) level with the PCM model in DMSO. According to the DP4+ probability analyses, (6aR*,8aS*,12aS*,12bS*)-**1** was assigned with a 100% probability (Table S5). Thus, the absolute configuration of **1** was suggested to be 6a*R*,8a*S*,12a*S*,12b*S*.

**Figure 5:**
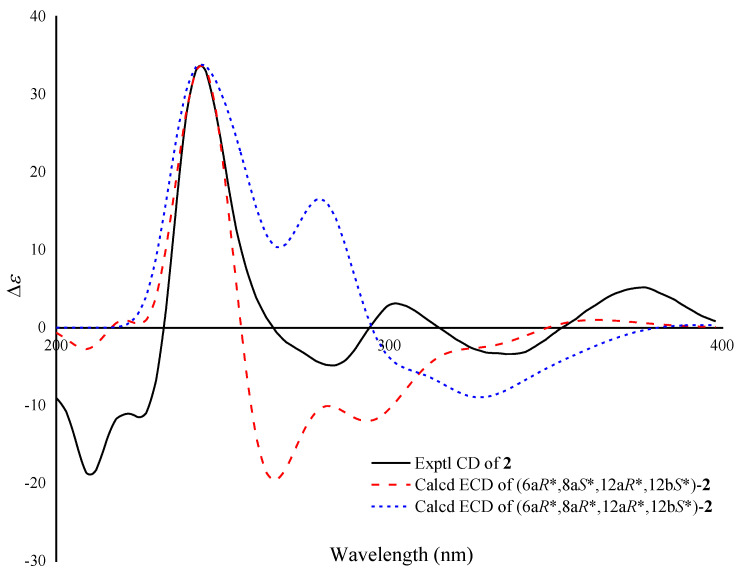
Calculated and experimental ECD spectra of **2**.

Compound **2** was isolated as a reddish powder. The HRESIMS showed [M + H]^+^ at *m/z* 375.1066, suggesting its molecular formula is C_19_H_18_O_8_, and corresponding to eleven degrees of unsaturation. Combination of the 1D NMR and HSQC spectra indicates 1 methyl (*δ*_C_ 23.6), 3 sp3 methylenes (*δ*_C_ 21.6, 28.2, and 41.8), 4 protonated sp2 carbons (*δ*_C_ 118.8, 122.8, 123.6, and 136.2), and 11 quaternary carbons including 3 non-protonated sp3 carbons (*δ*_C_ 75.4, 76.2, and 77.6), 5 non-protonated sp2 carbons (*δ*_C_ 109.5, 116.3, 133.0, 158.2, and 159.9), and 3 carbonyls (*δ*_C_ 192.8, 194.1, and 200.5) (Table 1). The structure of **2** was determined by careful analysis of its 2D NMR data (Figure 3), and its NMR data are quite similar to those of urdamycin J (**4**) [28] except for the presence of a hydroxyl group at C-6a in **2**, which was supported by the down-field shift of C-6a (*δ*_C_ 77.6, s) and the molecular formula. The theoretical NMR calculations and DP4+ probability analyses were employed to determine its final structure. According to the DP4+ probability analyses, (6aR*,8aS*,12aR*,12bS*)-**2** and (6aR*,8aR*,12aR*,12bS*)-**2** were assigned with a 23.61% and 67.24% probability, respectively (Table S6). The messages suggested that (6aR*,8aS*,12aR*,12bS*)-**2** or (6aR*,8aR*,12aR*,12bS*)-**2** was the correct relative structure for **2**. Finally, according to ECD calculations, the 6a*R*,8a*S*,12a*R* and 12b*S* absolute configuration for compound **2** was proposed. (Figure 5).

Compound **3** was also obtained as a reddish powder. Its molecular formula was established as C_19_H_14_O_5_ by the HRESIMS *m/z* 323.0908 [M + H]^+^ (calcd. for C_19_H_15_O_5_^+^, 323.0914), indicating 13 degrees of unsaturation. The ^1^H NMR spectrum showed signals of three aromatic protons (*δ*_H_ 7.71, dd, *J* = 8.4, 7.5 Hz; *δ*_H_ 7.48, dd, *J* = 7.5, 1.0 Hz; *δ*_H_ 7.31, dd, *J* = 8.4, 1.0 Hz), one olefinic proton (*δ*_H_ 6.58, s), two methylenes (*δ*_H_ 2.63 and *δ*_H_ 2.52), three methyl (*δ*_H_ 1.98, s), and three OH protons (*δ*_H_ 12.04, 9.80, and 8.40) (Table 1). The ^13^C NMR spectrum, in combination with the HSQC spectrum, indicates the presence of 1 methyl signal (*δ*_C_ 22.4), 2 methylene signals (*δ*_C_ 19.8 and 21.0), 4 methine signals (*δ*_C_ 115.7, 118.4, 123.2, and 136.2), 10 non-protonated sp2 carbons (*δ*_C_ 114.6, 121.2, 127.8, 129.2, 133.5, 139.2, 142.9, 145.3, 147.6, 159.8), and 2carbonyl carbons (*δ*_C_ 183.8 and 188.2) (Table 1). Preliminary analyses of these spectroscopic features implied that **3** had the same tetracyclic skeleton as that of **1** and **2**. Detailed analysis of 2D NMR data suggested that the structure of **3** was similar to 3,8-dihydroxy-l-methylbenz[*a*]anthracene-7,12-quinone [29] except for the presence of a hydroxyl group at C-9, which was supported by the HRESIMS data and the HMBC correlation from 9-OH (*δ*_H_ 9.80) to C-9 (*δ*_C_ 139.2) (Figure 3). 

Compounds **4**–**6** were identified as previously reported structures of urdamycin J (**4**) [28], 8-hydroxy-rabelomycin (**5**) and (−)-tetrangomycin (**6**) [30,31] by comparing their spectroscopic data. All three compounds showed promising cytotoxic activity during in vitro tests [28,32,33,34]. 

All new compounds were investigated for their cytotoxicity against L-02 (human normal liver cells), MDA-MB-231 (breast cancer), K562 (human myeloleukemia cells), ASPC-1 (human pancreatic cancer cells), H69AR (multidrug resistant human small cell lung cancer cells), and H69 (human small cell lung cancer cells) cell lines in vitro. Adriamycin was used as positive control. As a result, compound **1** showed cytotoxicity toward five cancer cell lines and especially strongly inhibited H69AR and H69 cell lines with an IC_50_ values of 0.59 and 0.32 μM, respectively (Table 2). 

In addition, all new compounds were tested for their antimicrobial activity against seven bacteria, including *Bacillus subtilis*, *Proteus vulgaris*, *B. cereus*, *Escherichia coli*, *Mycobacterium phlei*, *Acinetobacter baumannii*, and MRSA (methicillin-resistant *Staphylococcus aureus*), in addition to one yeast strain *Candida albicans* using previously described minimum inhibitory concentration (MIC) assay method [35]. Ciprofloxacin and nystatin were used as positive controls for pathogenic bacteria and yeast, respectively. All compounds did not show significant inhibitory effect against the tested strains (MIC > 50 µg/mL).

## 3. Materials and Methods

### 3.1. General Experimental Procedures

NMR spectra were recorded on a JEOLJN M-ECP 600 spectrometer (JEOL, Tokyo, Japan) or Bruker Avance Neo 400 MHz (Bruker, Beijing, China) using tetramethylsilane as an internal standard. Specific rotations were obtained on a JASCO P-1020 digital polarimeter. UV spectra were recorded on HITACHI 5430. IR spectra were measured on a Bruker Tensor-27 spectrophotometer in KBr discs. HRESIMS were obtained on a Thermo Scientific LTQ Orbitrap XL mass spectrometer (Thermo Fisher Scientific, Waltham, MA, USA) or Micromass Q-TOF ULTIMA GLOBAL GAA076 LC mass spectrometer (Waters Corporation, Shanghai, China). A JASCO J-715 spectropolarimeter (JASCO, Tokyo, Japan) was used to obtain ECD spectra. Semipreparative HPLC was performed on an ODS column (YMC-Pack ODS-A, 10 × 250 mm, 5 μm, 3 mL/min).

### 3.2. Materials and Culture Conditions

The actinomycete strain, *Streptomyces* sp. XS-16, was isolated from marine sediment samples collected from the sea around Naozhou Island in Zhanjiang, Guangdong Province. The strain was identified by 16S ribosomal RNA sequences (GenBank No. OQ449572). The strain was incubated in 2216E agar (5 g peptone, 1 g yeast extract, 1 g glucose, 0.1 g FePO_4_, 20 g agar, per liter seawater) at 28 °C for 5 days for cultivation. For compound isolation, the strain was cultured in the fermentation medium (20 g glucose, 4 g yeast extract, 1 g beef extract, 20 g soluble starch, 5 g soybean, 15 g K_2_HPO_4_, 20 g CaCO_3_ per liter water) at 28 °C, 180 rpm for 8 days. The strain was deposited at the Marine Medicinal Bioresources Center, Ocean University of China, Qingdao, China.

### 3.3. Sequence Analysis of the SCrp Gene

The Crp gene was analyzed by Localblast with the reported Crp obtained in National Center for Biotechnology Information (NCBI). For the multiple sequence alignment analysis, the amino acid sequences of SCrp and other Crp homologues from different species retrieved from NCBI were aligned using the ClustalX software [36]. The phylogenetic analysis was conducted with the MEGA7 software [37]. The conserved domain of the SCrp protein was scanned by the InterProScan program [38].

### 3.4. Construction of the SCrp Expression Vector

The overexpression vector pSET152 which mainly contains a constitutive promoter ermE*, site-specific recombinant elements phage φC31 integrase and the attachment site *attP* gene, resistant *acc3(IV)* apramycin gene as selection markers was digested with restriction endonuclease BamHI. The SCrp gene was PCR-amplified (from genomic DNA of the wild-type strain *Streptomyces* sp. XS-16) using specific primers SCrp-F/R (Table S1). The PCR products SCrp gene was introduced into pSET152 vector to generate pSET152-SCrp using the ClonExpress Ultra One Step Cloning Kit C115 (Vazyme) (Figure S3). The recombinant vector was transformed into competent *E. coli* strain DH5α to extract plasmids for transformation.

### 3.5. Overexpression, Fermentation, and Isolation of Compounds ***1***–***7*** in Streptomyces sp. XS-16

The plasmid pSET152-SCrp were introduced into *Streptomyces* sp. XS-16, via conjugation from *E. coli* ET12567/pUZ8002 according to the literature procedure and incubated at 28 °C for around 4 days [39].

### 3.6. Transformants Screening

After conjugation, the transformants were passaged to 2216E plates with 100 µg/mL apramycin, respectively. Apramycin-resistant mutants were transferred onto new 2216E media containing 100 µg/mL apramycin for the second screening. The strains that were able to grow were subjected to further PCR analysis validation. The putative OE::SCrp mutants and the wild-type strain were cultured on 2216E for 5 days at 28 °C.

### 3.7. Fermentation

For small-scale analysis, the transconjugants and the control strain were inoculated into 100 mL fermentation medium in 500 mL Erlenmeyer flasks and incubated at 28 °C for 8 days, after which the cultures were extracted with the volume of ethyl acetate for 3 times. The organic phase was evaporated, and the residue was dissolved in MeOH, which was analyzed by HPLC, which indicated that the mutants showed an apparent change in metabolite production (Figure 1).

For compound isolation, the selected strain was initially handled as above. Then a large-scale fermentation was performed in 500 mL Erlenmeyer flasks (total 30 L). The broth was extracted 3 times with ethyl acetate to give a total of 90 L of extract solution. The organic phase was evaporated under reduced pressure to afford a crude residue (3.2 g).

### 3.8. Extraction and Purification

Metabolites were monitored by LC-MS. The crude extract from 30 L fermentation of *Streptomyces* sp. XS-16 was subjected to a C_18_ column using a stepped gradient elution of MeOH-H_2_O, yielding 7 subfractions (Fr.1–Fr.7, 1% to 100%). Fr.2 was purified by semi-preparative HPLC (30:70 MeOH-H_2_O, 3 mL/min) to obtain compound **4** (4 mg, *t*_R_= 15.1 min). Fr.3 was purified by semi-preparative HPLC (45:55 MeOH-H_2_O, 3 mL/min) to obtain two subfractions (Fr.3.1–Fr.3.2). Fr.3.1 and Fr.3.2 were purified by semi-preparative HPLC (60:40, 47:53, MeOH-H_2_O, 3 mL/min) to afford **2** (7 mg, *t*_R_ = 22.3 min) and **5** (4 mg, *t*_R_ = 16.9 min), respectively. Fr.4 was applied on a Sephadex LH-20 column and eluted with MeOH to obtain four subfractions (Fr.4.1–Fr.4.4). Compounds **1** (3 mg, *t*_R_ = 31.1 min) and **3** (7 mg, *t*_R_ = 34.3 min) were obtained by semi-preparative HPLC (46:54, 52:48, MeOH-H_2_O, 3 mL/min) in Fr.4.1 and Fr.4.3, respectively. Fr.6 was purified by semi-preparative HPLC (80:20 MeOH-H_2_O, 3 mL/min) to obtain **6** (5 mg, *t*_R_ = 36.3 min).

Angumycinone E (**1**): pale yellowish powder; ${\left[\alpha \right]}_{\;D}^{25}$ + 22.5 (*c* 0.03, MeOH); UV (DAD) *λ*max 210 nm, 235 nm, 364 nm; CD (MeOH) *λ*max (∆ε) 230 (16.32), 267 (−15.82), 324 (7.68); IR (KBr) *ν*_max_ 3392, 2935, 1699, 1655, 1603, 1578, 1455, 1381, 1358, 1291, 1253, 1205, 1182, 1131, 1106, 1048, 1027, 1005, 976, 898, 801, 767, 730 cm^−1^. ^1^H and ^13^C NMR data, see Table 1; HRESIMS *m/z* 355.0824 [M − H]^−^ (calcd. for C_19_H_15_O_7_, 355.0823).
 
Angumycinone F (**2**): reddish powder; ${\left[\alpha \right]}_{\;D}^{25}$ + 19.5 (*c* 0.03, MeOH); UV (DAD) *λ*max 221 nm, 270 nm, 421 nm; CD (MeOH) *λ*max (∆ε) 245 (33.27), 282 (−5.17), 301 (−3.54); IR (KBr) *ν*_max_ 2922, 2852.87,1683, 1209, 1187,1134, 1079, 1033 cm^−1^. ^1^H and ^13^C NMR data, see Table 1; HRESIMS *m/z* 375.1066 [M + H]^+^ (calcd. for C_19_H_19_O_8_, 375.1074).
 
Kanglemycin E (**3**): reddish powder; ${\left[\alpha \right]}_{\;D}^{25}$ − 15.2 (*c* 0.03, MeOH); UV (DAD) *λ*max 205 nm, 234 nm, 363 nm; IR (KBr) *ν*_max_ 3415.88, 2921.14, 1683.06, 1639.65, 1457.12, 1384.38, 1283.86, 1207.35, 1139.29, 1027.47, 838.46, 801.60, 722.46 cm^−1^. ^1^H and ^13^C NMR data, see Table 1; HRESIMS *m*/*z* 323.0908 [M + H]^+^ (calcd. for C_19_H_15_O_5_, 323.0914).

### 3.9. Computational Section

#### 3.9.1. NMR Calculations

Conformation searches based on molecular mechanics with MMFF force fields were performed for stereoisomers to get stable conformers within 20 kJ/mol. All conformers were further optimized by the density functional theory method at the B3LYP/6-31G(d) level by the Gaussian 16 program package. Gauge Independent Atomic Orbital (GIAO) calculations of their ^1^H and ^13^C NMR chemical shifts using density functional theory (DFT) at the mPW1PW91/6-311+G(d,p) level with the PCM model in DMSO. The calculated NMR data of these conformers were averaged according to the Boltzmann distribution theory and their relative Gibbs free energy. The ^1^H and ^13^C NMR chemical shifts for TMS were also calculated by the same procedures and used as the reference. After calculation, the experimental and calculated data were evaluated by linear correlation coefficients (*R*^2^) and the improved probability DP4+ method [40].

#### 3.9.2. ECD Calculations

All stable conformers were further optimized by the density functional theory method at the B3LYP/6-31G(d) level by the Gaussian 16 program package. The ECD were calculated using density functional theory (TDDFT) at the B3LYP/6-31+G(d,p) level in methanol with IEFPCM model. The calculated ECD curves were all generated using SpecDis 1.71 program package (version number, manufacturer’s name, city, and country Version 1.71, SpecDis, Berlin, Germany, https://specdis-software.jimdo.com, accessed on 25 February 2023) and the calculated ECD data of all conformers were Boltzmann averaged by Gibbs free energy.

### 3.10. Cytotoxicity Assay

Cytotoxic activities of new compounds were evaluated against K562 (using the MTT method), MDA-MB-231, L-02, H69AR, and ASPC-1 (using the SRB method) cell lines. Adriamycin (ADM) was used as a positive control. The detailed methodologies for biological testing have been described in previous reports [41,42].

### 3.11. Antimicrobial Activity

Antimicrobial activities of new compounds against seven bacteria, including *B. subtilis*, *P. vulgaris*, *B. cereus*, *E. coli*, *M. phlei*, *A. baumannii*, and MRSA, in addition to one fungal strain *C. albicans* were carried out by using minimum inhibitory concentration (MIC) assay method as previously reported [35]. Ciprofloxacin was used as a positive control.

## 4. Conclusions and Outlook

Crp is a global transcription regulator that controls transcription initiation and widely affects the primary and secondary metabolism of bacteria. By overexpression of the native Crp homologue SCrp in the marine-derived strain of *Streptomyces* sp. XS-16, we discovered three undescribed angucycline derivatives **1**–**3**. Structurally, compounds **1** and **2** are highly oxidized angucyclines, and they are isomerized at C-6a, C-12a, and C-12b. The angucycline structures have been reported to be derived from type II polyketide biosynthetic pathway [43]. One type II PKS gene cluster (cluster 1, Figure S1) has been discovered from the genome of *Streptomyces* sp. XS-16. Biosynthetic study of above angucycline derivatives is currently in progress.

The isolated compounds **1**–**3** were evaluated for their cytotoxic and antimicrobial activities in vitro. In addition, compound **1** showed inhibitory activities against five tumor cell lines with IC_50_ values ranging from 0.32 to 5.33 μM. So far, this is the first report on the application of the global regulator Crp in marine-derived *Streptomyces* species. Specifically, Crp could upregulate the expression of genes involved in angucyclines biosynthesis. In addition, Crp is also a multifunctional regulator that modulates primary metabolism and enhances precursor flux to secondary metabolite biosynthesis. The above results support Crp as a useful tool exploiting the metabolic potential of marine-derived *Streptomyces* and developing the structural diversity of secondary metabolites.

## Figures and Tables

**Table 1:** ^1^H and ^13^C NMR Spectroscopic Data for **1**–**3** in DMSO-*d*_6_.

No.	1 ^a^		2 ^b^		3 ^b^	
	*δ*_C_, Type	*δ*_H_ (*J* in Hz)	*δ*_C_, Type	*δ*_H_ (*J* in Hz)	*δ*_C_, Type	*δ*_H_ (*J* in Hz)
1	189.8, C	–	192.8, C	–	183.8, C	–
1a	131.9, C	–	133.0, C	–	133.5, C	–
2	118.8, CH	7.41, d, (7.6, 1.0)	118.8, CH	7.61, dd, (7.5, 1.1)	118.4, CH	7.48, dd, (7.5, 1.0)
3	136.7, CH	7.71, dd (8.4, 7.6)	136.2, CH	7.76, dd (8.4, 7.5)	136.2, CH	7.71, dd, (8.4, 7.5)
4	124.3, CH	7.34, dd (8.4, 1.0)	123.6, CH	7.35, dd (8.4, 1.1)	123.2, CH	7.31, dd, (8.4, 1.0)
5	159.8, C	–	159.9, C	–	159.8, C	–
5a	114.3, C	–	116.3, C	–	114.6, C	–
6	194.0, C	–	194.1, C	–	188.2, C	–
6a	72.8, C	–	77.6, C	–	145.3, C	–
7α	17.4, CH_2_	2.80, m	21.6, CH_2_	2.28, m	21.0, CH_2_	2.63, m
7β		2.27, m		1.77, m		
8α	27.5, CH_2_	1.82, m	28.2, CH_2_	2.01, m	19.8, CH_2_	2.52, m
8β		1.49, m		1.61, m		
8a	63.6, C	–	75.4, C	–	147.6, C	–
9α	41.5, CH_2_	2.70, m	41.8, CH_2_	2.90, d, (18.0)	139.2, C	–
9β		2.24, m		2.07, d (18.0)		
10	159.2, C	–	158.2, C	–	127.8, C	–
11	121.7, CH	5.91, s	122.8, CH	5.72, s	115.7, CH	6.58, s
12	195.5, C	–	200.5, C	–	129.2, C	–
12a	109.5, C	–	109.5 C	–	121.2, C	–
12b	75.2, C	–	76.2, C	–	142.9, C	–
13	23.4, CH_3_	1.86, s	23.6, CH_3_	1.94, s	22.4, CH_3_	1.98, s
5-OH	–	11.16, s	–	11.11, s	–	12.04, s
9-OH	–	–	–	–	–	9.80, s
10-OH	–	–	–	–	–	8.40, s

^a^ Assignment at 600 MHz/150 MHz, ^b^ Assignment at 500 MHz/125 MHz.

**Table 2:** Cytotoxicity of **1**–**3** (IC_50_, μM).

Compounds	Inhibition Ratio	IC_50_ (μM)				
	L-02	MDA-MB-231	K562	ASPC-1	H69AR	H69
**1**	10.01%	4.91	4.85	5.33	0.59	0.32
**2**	3.83%	>50	>25	>50	>50	>50
**3**	0.15%	>50	>50	>50	>50	>50
Adriamycin	89.62%	0.31	0.24	0.22	0.52	0.36

## Data Availability

The data presented in this study are available in this article and the Supplementary Materials.

## Supplementary Materials

The following supporting information can be downloaded at: https://www.mdpi.com/article/10.3390/md21040240/s1: Figure S1: AntiSMASH analysis of the genome of the strain *Streptomyces* sp. XS-16; Figure S2. Map of the vector SCrp overexpression plasmid pSET152-SCrp. Figure S3: PCR analysis for confirming the plasmid construction; Figure S4. Comparison of the calculated ECD spectra for 1a, 1b, 1c, and 1d with the experimental spectrum of 1 in methanol with PCM model. Figure S5. ^13^C NMR calculation results of two possible isomers of 1. Figure S6. Comparison of the calculated ECD spectra for 2a and 2b with the experimental spectrum of 2 in methanol with PCM model. Figures S7–S13: NMR, HRESIMS, and IR spectra of compound **1**; Figures S14–S20: NMR, HRESIMS, and IR spectra of compound **2**; Figures S21–S28: NMR, HRESIMS, and IR spectra of compound **3**; Table S1: The primers used in this study; Table S2. The sequence of SCrp. (5′ to 3′); Table S3. Calculated ^13^C NMR results for 1a. Table S4. Calculated ^13^C NMR results for 2d. Table S5. DP4+ analysis results of 1a (Isomer 1) and 1d (Isomer 2). Table S6. DP4+ analysis results of 2a (Isomer 1), 2b (Isomer 2), 2c (Isomer 3), 2d (Isomer 4), 2e (Isomer 5), 2f (Isomer 6), 2g (Isomer 7), and 2h (Isomer 8).
