# Supplementary material for: Overexpression of Global Regulator SCrp Leads to the Discovery of New Angucyclines in Streptomyces sp. XS-16
Source: Mar Drugs. 2023 Apr 14;21(4):240. doi: 10.3390/md21040240 (PMC10146017; doi:10.3390/md21040240)

Contents

Section S1. Construction section of the plasmid pSET152-SCrp 2

Section S2. Calculational details for **1** 5

S2.1. Computational details for compound **1** (ECD) 5

S2.2. Computational details for compound **1** (NMR) 6

Section S3. Calculational details for **2** 11

S3.1. Computational details for compound **2** (NMR) 11

S3.2. Computational details for compound **2** (ECD) 13

Section S4. NMR, HRESIMS, and IR spectra of **1** 14

Section S5. NMR, HRESIMS, and IR spectra of **2** 18

Section S6. NMR, HRESIMS, and IR spectra of **3** 22

Section S1. Construction section of the plasmid pSET152-SCrp

Table S1. The primers used in this study. (5' to 3')

Primers	Sequences
SCrp-F	ACAATCGTGCCGGTTGGTAGGATCCgtggacgacgttctgcggcg
SCrp-R	AGCTTGGGCTGCAGGTCGACTCTAGAtcagcgcgagcgcttcgcc
YZ-SCrp-F	GAGTTCGAGGTACGCGGCTTG
YZ-SCrp-R	CCTCTTCGCTATTACGCCAGCTG

Note: The homologous arms are represented by lowercase letters.

Figure S1. AntiSMASH analysis of the genome of the strain *Streptomyces* sp. XS-16. The predicted 29 gene clusters include 5 PKS, 6 NRPS, 5 Terpene, 1 PKS-NRPS hybrids, 2 Bacteriocin, 10 Other clusters.

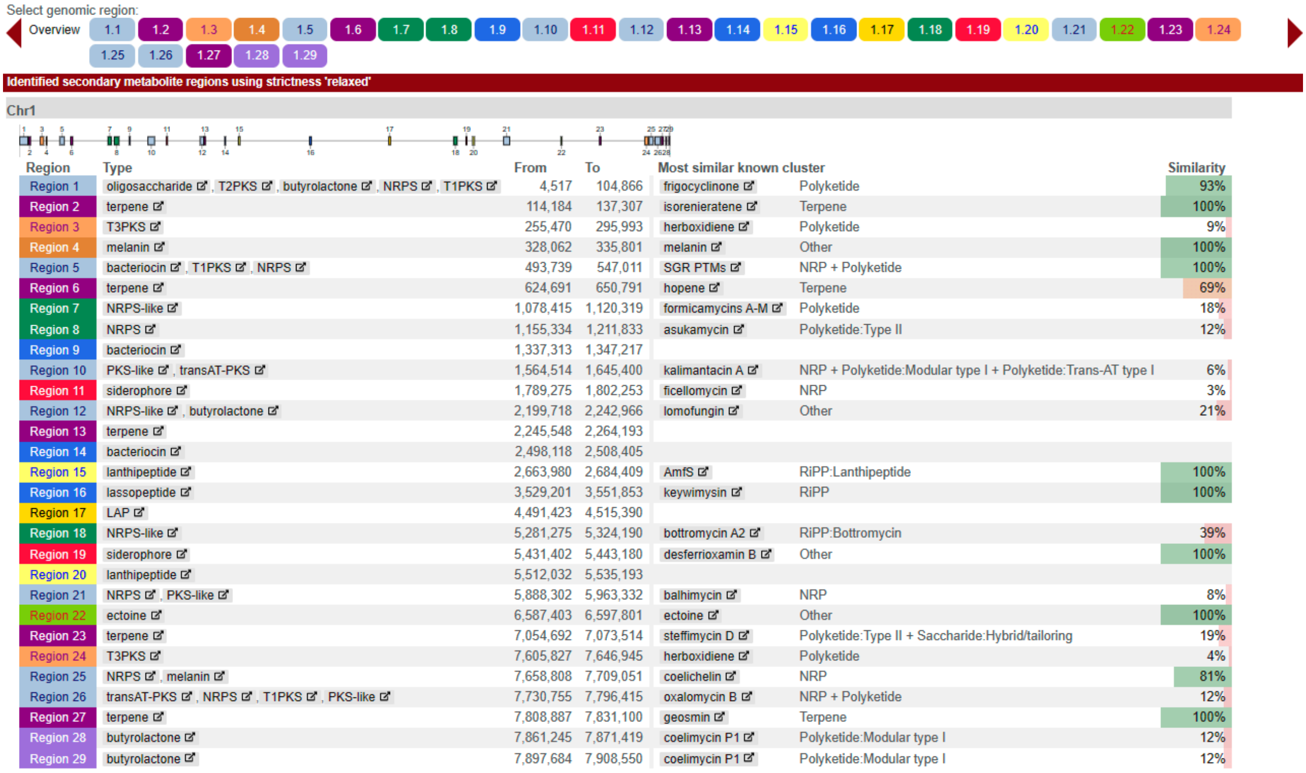
Select genomic region:
Overview 1.1 1.2 1.3 1.4 1.5 1.6 1.7 1.8 1.9 1.10 1.11 1.12 1.13 1.14 1.15 1.16 1.17 1.18 1.19 1.20 1.21 1.22 1.23 1.24 1.25 1.26 1.27 1.28 1.29

Identified secondary metabolite regions using strictness 'relaxed'

Chr1

Region	Type	From	To	Most similar known cluster		Similarity
Region 1	oligosaccharide, T2PKS, butyrolactone, NRPS, T1PKS	4,517	104,866	frigocyclinone	Polyketide	93%
Region 2	terpene	114,184	137,307	isorenieratene	Terpene	100%
Region 3	T3PKS	255,470	295,993	herboxidiene	Polyketide	9%
Region 4	melanin	328,062	335,801	melanin	Other	100%
Region 5	bacteriocin, T1PKS, NRPS	493,739	547,011	SGR PTMs	NRP + Polyketide	100%
Region 6	terpene	624,691	650,791	hopene	Terpene	69%
Region 7	NRPS-like	1,078,415	1,120,319	formicamycins A-M	Polyketide	18%
Region 8	NRPS	1,155,334	1,211,833	asukamycin	Polyketide:Type II	12%
Region 9	bacteriocin	1,337,313	1,347,217			
Region 10	PKS-like, transAT-PKS	1,564,514	1,645,400	kalimantacin A	NRP + Polyketide:Modular type I + Polyketide:Trans-AT type I	6%
Region 11	siderophore	1,789,275	1,802,253	ficellomycin	NRP	3%
Region 12	NRPS-like, butyrolactone	2,199,718	2,242,966	lomofungin	Other	21%
Region 13	terpene	2,245,548	2,264,193			
Region 14	bacteriocin	2,498,118	2,508,405			
Region 15	lanthipeptide	2,663,980	2,684,409	AmfS	RiPP:Lanthipeptide	100%
Region 16	lassopeptide	3,529,201	3,551,853	keywimysin	RiPP	100%
Region 17	LAP	4,491,423	4,515,390			
Region 18	NRPS-like	5,281,275	5,324,190	bottromycin A2	RiPP:Bottromycin	39%
Region 19	siderophore	5,431,402	5,443,180	desferrioxamin B	Other	100%
Region 20	lanthipeptide	5,512,032	5,535,193			
Region 21	NRPS, PKS-like	5,888,302	5,963,332	balhimycin	NRP	8%
Region 22	ectoine	6,587,403	6,597,801	ectoine	Other	100%
Region 23	terpene	7,054,692	7,073,514	steffimycin D	Polyketide:Type II + Saccharide:Hybrid/tailoring	19%
Region 24	T3PKS	7,605,827	7,646,945	herboxidiene	Polyketide	4%
Region 25	NRPS, melanin	7,658,808	7,709,051	coelichelin	NRP	81%
Region 26	transAT-PKS, NRPS, T1PKS, PKS-like	7,730,755	7,796,415	oxalomycin B	NRP + Polyketide	12%
Region 27	terpene	7,808,887	7,831,100	geosmin	Terpene	100%
Region 28	butyrolactone	7,861,245	7,871,419	coelimycin P1	Polyketide:Modular type I	12%
Region 29	butyrolactone	7,897,684	7,908,550	coelimycin P1	Polyketide:Modular type I	12%

Table S2. The sequence of SCrp. (5’ to 3’)

Name	Sequence
SCrp	GTGGACGACGTTCTGCGGCGCGCCCCGCTTTTCGCGGCGCTCGATGATGAGCAGGCCGCGG<br>AGCTCCGCGCCTCGATGAGTGAGGTGACCCTCGCGCGCGGCGACGCGCTTTTCCATGAGGG<br>CGACCAGGGTGACCGCCTGTACGTGGTCACCGAGGGCAAGGTGAAGCTCCACCGCACCTC<br>GCCCGACGGGCGCGAGAACATGCTGGCCGTGCTCGGTCCCGGCGAGCTGATCGGTGAGCT<br>CTCGCTCTTCGACCCCGGCCCGCGTACGGCGACCGCCTCCGCGCTGACCGAGGTCAAGCTC<br>CTCGGCCTCGGCCACGGCGACCTGCAGCCCTGGCTCAACGCCCGGCCCGAGGTCGCCACCG<br>CGCTGCTGCGCGCGGTCGCCCGGCGCCTGCGCAAGACCAACGACCAGATGTCCGACCTGG<br>TCTTCTCCGACGTGCCGGGCCGTGTCGCCCGCGCCCTCCTGGACCTGTCGCGCCGCTTCGGC<br>GTCCAGTCGGAGGAAGGAATCCACGTCGTGCACGACCTCACCCAGGAGGAGCTGGCCCAG<br>CTGGTCGGCGCCTCCCGCGAGACGGTCAACAAGGCCCTCGCGGACTTCGCGGGCCGCGGC<br>TGGCTGCGCCTGGAGGCCCGCGCGGTCATCCTGCTGGACGTGGAGCGCCTGGCGAAGCGC<br>TCGCGCTGA

Note: The homologous arms are represented by lowercase letters.

Figure S2. Map of the vector SCrp overexpression plasmid pSET152-SCrp.

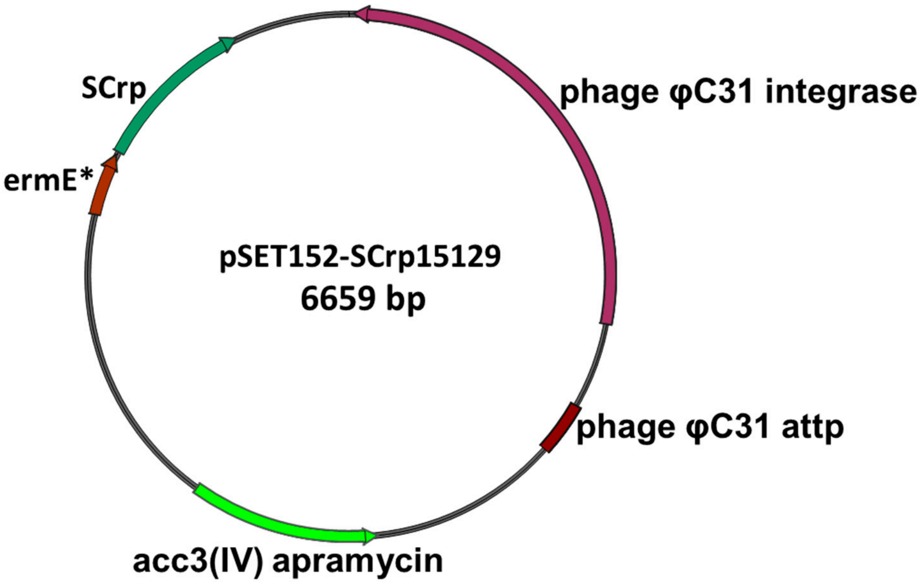

Figure S3. PCR analysis for confirming the plasmid construction. The results showed that mutants 1-5 were desired.

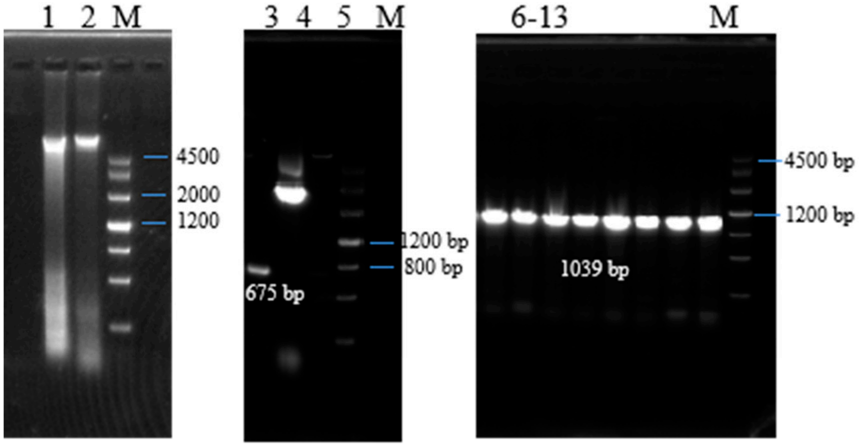

Notes:
1-2： the genome of XS-16;
3： pSET152-XS-16-crp after purification
4： pSET152-ermE*;
5： pSET152-ermE*(BamHI)
6-13: pSET152-SCrp mutants
M: markerIII

Section S2. Calculational details for 1

S2.1. Computational details for compound 1 (ECD)

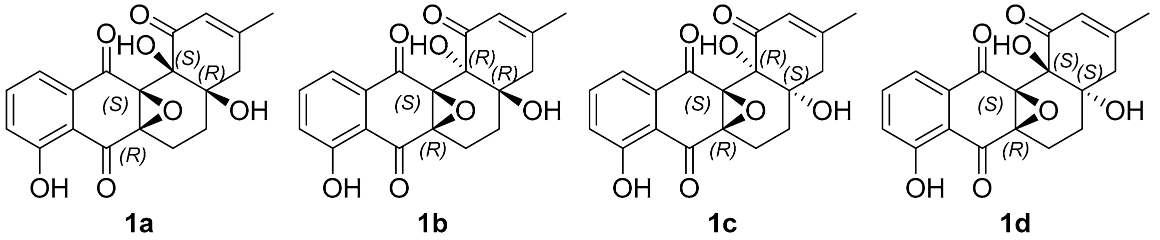

Conformation search based on molecular mechanics with MMFF force fields (Spartan′14, Wavefunction, Inc.) were performed for **1a**, **1b**, **1c**, and **1d** gave 3, 4, 3, and 2 stable conformers within 20 kJ/mol, respectively. All these conformers were further optimized by the density functional theory method at the B3LYP/6-31G(d) level by Gaussian 16 program package. The ECD of stable conformers with populations higher than 1% were calculated using density functional theory (TDDFT) at B3LYP/6-31+G(d) level in methanol with IEFPCM model. The calculated ECD curves were all generated using SpecDis 1.71 with $\sigma = 0.30$ eV, and UV shift -18 nm, respectively.

Figure S4. Comparison of the calculated ECD spectra for **1a**, **1b**, **1c**, and **1d** with the experimental spectrum of **1** in methanol with PCM model.

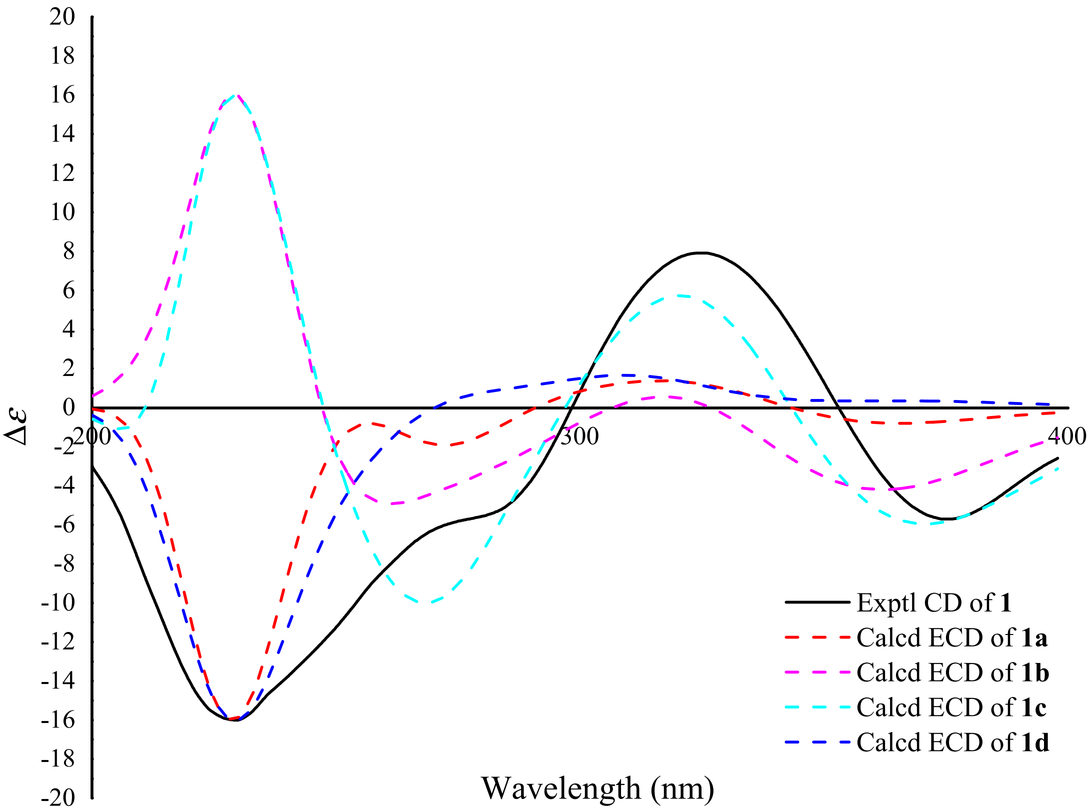

S2.2. Computational details for compound 1 (NMR)

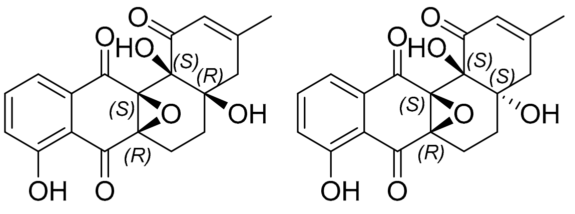

Conformation search based on molecular mechanics with MMFF force fields (Spartan′14, Wavefunction, Inc.) were performed for **1a** and **1d** gave 3 and 2 stable conformers within 20 kJ/mol, respectively. All these conformers were further optimized by the density functional theory method at the B3LYP/6-31G(d) level by Gaussian 16 program package. Gauge Independent Atomic Orbital (GIAO) calculations of their ^{1}H and ^{13}C NMR chemical shifts of stable conformers with populations higher than 1% using density functional theory (DFT) at the mPW1PW91/6-311+G(d,p) level with the PCM model in DMSO. The calculated NMR data of these conformers were averaged according to the Boltzmann distribution theory and their relative Gibbs free energy. The ^{1}H and ^{13}C NMR chemical shifts for TMS were also calculated by the same procedures and used as the reference. After calculation, the experimental and calculated data were evaluated by linear correlation coefficients (R^2) and the improved probability DP4+ method.

Table S3. Calculated ^{13}C NMR results for **1a**

No.	**1aA**	**1aB**	**1aC**	$\delta_{Calcd}{}^{a}$	δ_{Exp}	$\delta_{Corr}{}^{b}$	Relative errors^{c}
1	51.40	51.28	50.29	137.7	132.4	127.6	4.8
2	69.33	69.42	69.74	119.8	114.8	110.6	4.2
3	-18.87	-19.40	-17.74	208.0	194.5	194.2	0.3
4	118.32	119.00	115.53	70.8	71.6	64.3	7.3
5	118.37	118.53	114.07	70.7	64.1	64.2	-0.1
6	-15.08	-15.49	-7.44	204.2	192.1	190.6	1.5
7	61.45	61.45	61.58	127.6	119.2	118.1	1.1
8	17.30	17.46	17.85	171.8	160.3	159.9	0.4
9	54.53	54.76	56.38	134.6	125.2	124.7	0.5
10	42.57	42.64	42.17	146.5	137.2	136.0	1.2
11	166.64	166.60	167.09	22.5	17.9	18.5	-0.6
12	157.21	154.37	156.70	31.9	27.9	27.4	0.5
13	108.32	107.88	104.53	80.8	63.5	73.7	-10.2

14	104.65	104.57	107.52	84.4	75.7	77.2	-1.5
15	-19.37	-19.97	-18.99	208.5	196.0	194.6	1.4
16	59.12	59.29	58.49	130.0	122.2	120.3	1.9
17	140.63	140.68	142.65	48.5	41.9	43.1	-1.2
18	8.63	10.54	13.45	180.5	156.9	168.1	-11.2
19	160.63	160.93	161.05	28.5	23.9	24.2	-0.3
Population	99.94%	0.00%	0.06%			RMSD	4.2

[a]Weighted average from the calculated shifts; [b]Obtained by linear fit δexp versus δcalcd; [c]$\Delta\delta = \delta_{exp} - \delta_{corr}$.

Table S4. Calculated ^{13}C NMR results for **1d**

No.	**1dA**	**1dB**	$\delta_{Calcd}{}^{a}$	δ_{Exp}	$\delta_{Corr}{}^{b}$	Relative errors[c]
1	50.20	50.50	138.6	132.4	129.1	3.3
2	68.79	68.79	120.3	114.8	111.6	3.2
3	-19.27	-19.11	208.2	194.5	195.4	-0.9
4	116.89	116.76	72.3	71.6	65.9	5.7
5	117.87	118.10	71.0	64.1	64.7	-0.6
6	-11.99	-12.14	201.2	192.1	188.7	3.4
7	61.95	61.88	127.2	119.2	118.2	1.0
8	17.22	17.24	171.9	160.3	160.7	-0.4
9	55.20	55.03	134.0	125.2	124.7	0.5
10	42.45	42.50	146.6	137.2	136.7	0.5
11	165.06	165.56	23.6	17.9	19.5	-1.6
12	158.17	159.27	30.0	27.9	25.6	2.3
13	108.01	109.14	80.1	63.5	73.3	-9.8
14	106.66	105.83	83.2	75.7	76.2	-0.5
15	-18.45	-18.72	207.8	196.0	195.0	1.0
16	58.09	57.12	131.9	122.2	122.6	-0.4
17	142.12	142.79	46.4	41.9	41.2	0.7
18	15.82	13.19	175.6	156.9	164.3	-7.4
19	161.29	160.90	28.1	23.9	23.8	0.1

Population	12.27%	87.73%	RMSD	3.5

[a]Weighted average from the calculated shifts; [b]Obtained by linear fit δexp versus δcalcd; [c]$\Delta\delta = \delta_{exp} - \delta_{corr}$.

Figure S5. ^{13}C NMR calculation results of two possible isomers of **1**. (a) Linear correlation plots of predicted versus experimental ^{13}C NMR chemical shifts. (b) Relative errors between the predicted ^{13}C NMR chemical shifts of two potential structures and recorded ^{13}C NMR data.

1a A

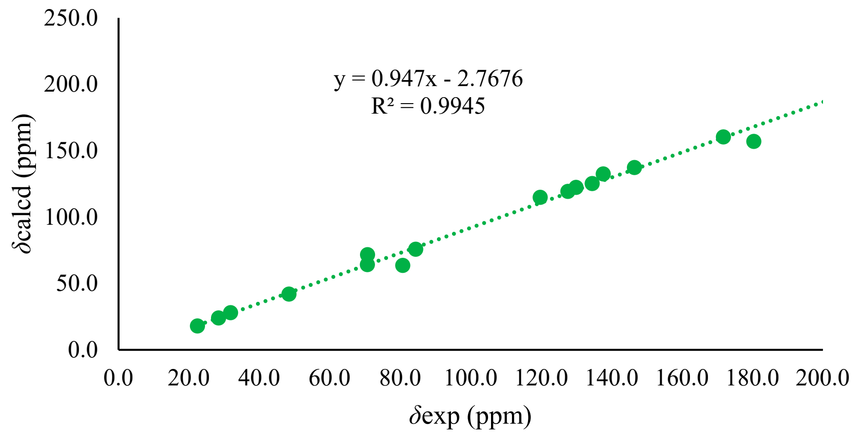

1a B

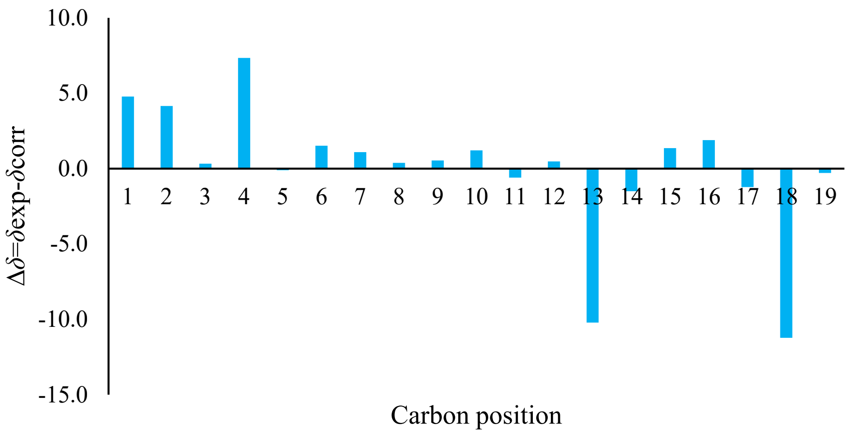

1d A

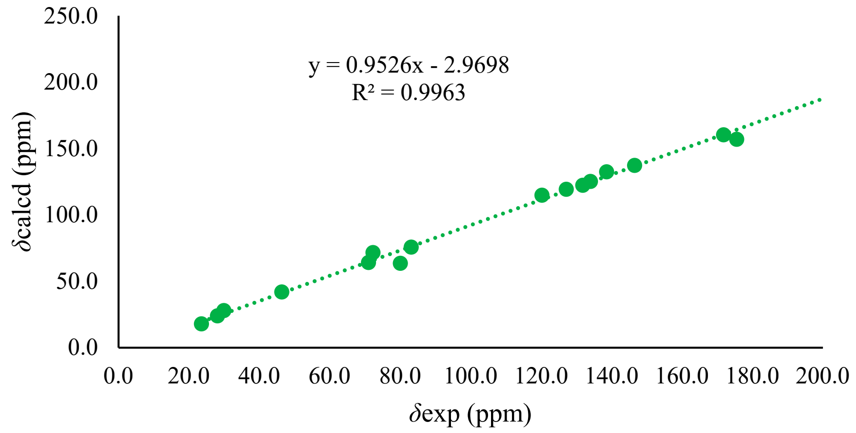

1d B

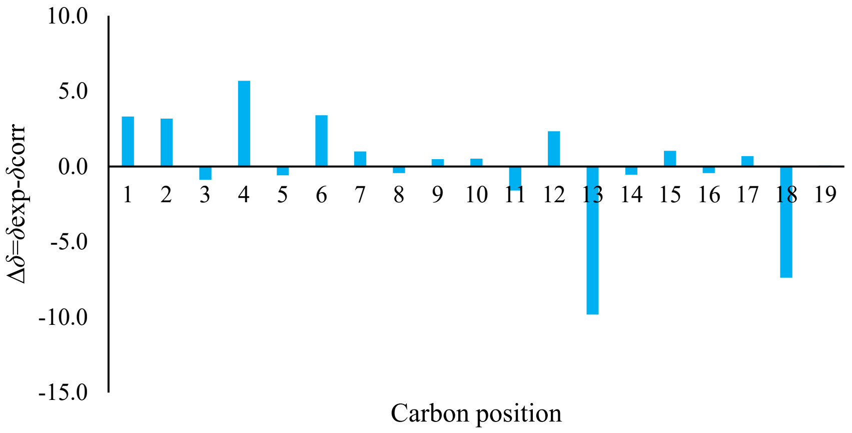

Table S5. DP4+ analysis results of **1a** (Isomer 1) and **1d** (Isomer 2)

	A	B	C	D	E	F	G	H
1	Functional		Solvent?		Basis Set		Type of Data	
2	mPW1PW91		PCM		6-311+G(d,p)		Shielding Tensors	
3								
12			DP4+	0.00%	100.00%	–	–	–
14	Nuclei	sp2?	Experimenta	Isomer 1	Isomer 2	Isomer 3	Isomer 4	Isomer 5
15	C	x	132.4	51.4	50.5			
16	C	x	114.8	69.3	68.8			
17	C	x	194.5	-18.9	-19.1			
18	C		71.6	118.3	116.8			
19	C		64.1	118.4	118.1			
20	C	x	192.1	-15.1	-12.1			
21	C	x	119.2	61.5	61.9			
22	C	x	160.3	17.3	17.2			
23	C	x	125.2	54.5	55.1			
24	C	x	137.2	42.6	42.5			
25	C		17.9	166.6	165.5			
26	C		27.9	157.2	159.1			
27	C		63.5	108.3	109.0			
28	C		75.7	104.6	105.9			
29	C	x	196	-19.4	-18.7			
30	C	x	122.2	59.1	57.2			
31	C		41.9	140.6	142.7			
32	C	x	156.9	8.6	13.5			
33	C		23.9	160.6	161.0			

	A	B	C	D	E	F	G	H
1	Functional		Solvent?		Basis Set		Type of Data	
2	mPW1PW91		PCM		6-311+G(d,p)		Shielding Tensors	
3								
4			Isomer 1	Isomer 2	Isomer 3	Isomer 4	Isomer 5	Isomer 6
5	sDP4+ (H data)		–	–	–	–	–	–
6	sDP4+ (C data)		6.00%	94.00%	–	–	–	–
7	sDP4+ (all data)		6.00%	94.00%	–	–	–	–
8	uDP4+ (H data)		–	–	–	–	–	–
9	uDP4+ (C data)		0.00%	100.00%	–	–	–	–
10	uDP4+ (all data)		0.00%	100.00%	–	–	–	–
11	DP4+ (H data)		–	–	–	–	–	–
12	DP4+ (C data)		0.00%	100.00%	–	–	–	–
13	DP4+ (all data)		0.00%	100.00%	–	–	–	–

Section S3. Calculational details for 2

S3.1. Computational details for compound 2 (NMR)

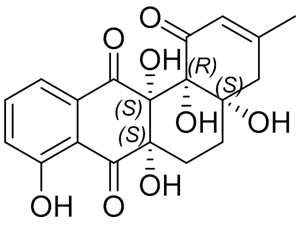

2a

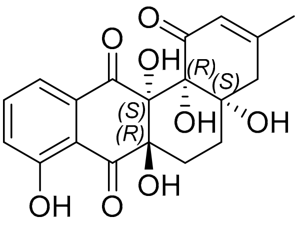

2b

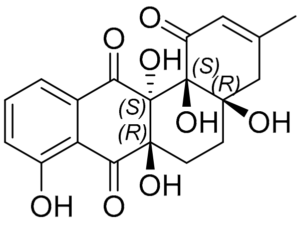

2c

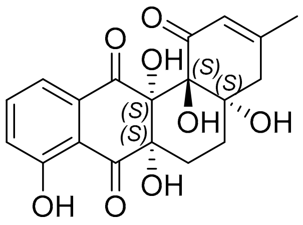

2d

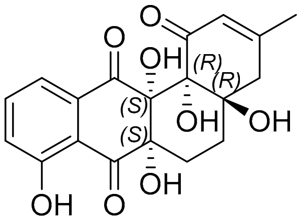

2e

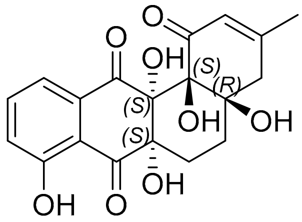

2f

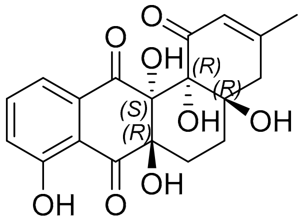

2g

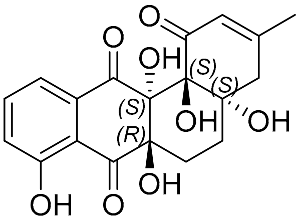

2h

Conformation search based on molecular mechanics with MMFF force fields (Spartan′14, Wavefunction, Inc.) were performed for **2a**, **2b**, **2c**, **2d**, **2e**, **2f**, **2g** and **2h** gave 5, 3, 1, 4, 2, 1, 2 and 4 stable conformers within 20 kJ/mol, respectively. All these conformers were further optimized by the density functional theory method at the B3LYP/6-31G(d) level by Gaussian 16 program package. Gauge Independent Atomic Orbital (GIAO) calculations of their ^{1}H and ^{13}C NMR chemical shifts of stable conformers with populations higher than 1% using density functional theory (DFT) at the mPW1PW91/6-311+G(d,p) level with the PCM model in DMSO. The calculated NMR data of these conformers were averaged according to the Boltzmann distribution theory and their relative Gibbs free energy. The ^{1}H and ^{13}C NMR chemical shifts for TMS were also calculated by the same procedures and used as the reference. After calculation, the experimental and calculated data were evaluated by linear correlation coefficients (R^2) and the improved probability DP4+ method.

Table S6. DP4+ analysis results of **2a** (Isomer 1), **2b** (Isomer 2), **2c** (Isomer 3), **2d** (Isomer 4), **2e** (Isomer 5), **2f** (Isomer 6), **2g** (Isomer 7), and **2h** (Isomer 8)

	A	B	C	D	E	F	G	H	I	J	K
1	Functional		Solvent?		Basis Set		Type of Data				
2	mPW1PW91		PCM		6-311+G(d, p)		Shielding Tensors				
3											
12			DP4+	0. 00%	23. 61%	0. 00%	0. 00%	0. 00%	0. 00%	67. 24%	9. 15%
14	Nuclei	sp2?	xperimenta	Isomer 1	Isomer 2	Isomer 3	Isomer 4	Isomer 5	Isomer 6	Isomer 7	Isomer 8
15	C	x	136. 2	42. 0	43. 4	43. 3	43. 3	41. 4	42. 0	42. 6	42. 8
16	C	x	123. 6	56. 2	54. 4	55. 5	55. 3	55. 4	54. 7	55. 7	55. 6
17	C	x	159. 9	18. 5	17. 8	18. 3	18. 2	18. 5	18. 7	17. 3	18. 1
18	C	x	116. 3	69. 9	67. 3	67. 7	64. 0	69. 4	68. 8	67. 9	67. 7
19	C	x	133	46. 1	50. 8	49. 0	48. 1	48. 7	49. 1	50. 4	47. 8
20	C	x	118. 8	62. 0	61. 1	61. 6	62. 3	60. 8	60. 2	61. 8	62. 3
21	C	x	194. 1	-25. 7	-22. 6	-23. 2	-18. 5	-25. 6	-26. 2	-21. 9	-21. 2
22	C		77. 6	104. 5	106. 3	104. 8	103. 3	101. 9	102. 2	106. 9	103. 3
23	C		76. 2	98. 0	108. 5	105. 4	98. 8	103. 5	106. 2	110. 1	103. 9
24	C	x	192. 8	-18. 4	-19. 4	-25. 1	-21. 5	-17. 1	-20. 5	-17. 6	-24. 2
25	C		21. 6	151. 1	163. 4	159. 0	159. 7	151. 1	150. 9	156. 9	162. 2
26	C		28. 2	153. 4	158. 4	159. 3	153. 0	155. 8	156. 0	155. 6	156. 5
27	C		75. 4	112. 9	104. 7	107. 4	108. 1	107. 4	107. 2	106. 7	106. 6
28	C		109. 5	111. 8	105. 4	103. 6	105. 2	111. 4	108. 7	103. 0	106. 5
29	C		41. 8	139. 8	140. 8	141. 0	142. 6	141. 8	141. 7	141. 9	142. 0
30	C	x	158. 2	11. 0	8. 0	7. 0	7. 8	5. 4	6. 7	13. 9	9. 6
31	C	x	122. 8	56. 6	57. 6	57. 2	59. 1	58. 2	57. 5	56. 3	59. 0
32	C	x	200. 5	-20. 7	-20. 8	-19. 8	-14. 5	-19. 4	-20. 2	-16. 3	-23. 9
33	C		23. 6	159. 9	160. 3	160. 3	159. 8	158. 9	159. 9	161. 2	160. 7

	A	B	C	D	E	F	G	H	I	J
1	Functional		Solvent?		Basis Set		Type of Data			
2	mPW1PW91		PCM		6-311+G(d, p)		Shielding Tensors			
3										
4			Isomer 1	Isomer 2	Isomer 3	Isomer 4	Isomer 5	Isomer 6	Isomer 7	Isomer 8
5	sDP4+ (H data)		-	-	-	-	-	-	-	-
6	sDP4+ (C data)		0. 00%	8. 58%	0. 60%	0. 34%	0. 00%	0. 02%	0. 08%	90. 39%
7	sDP4+ (all data)		0. 00%	8. 58%	0. 60%	0. 34%	0. 00%	0. 02%	0. 08%	90. 39%
8	uDP4+ (H data)		-	-	-	-	-	-	-	-
9	uDP4+ (C data)		0. 00%	0. 31%	0. 00%	0. 00%	0. 00%	0. 00%	99. 68%	0. 01%
10	uDP4+ (all data)		0. 00%	0. 31%	0. 00%	0. 00%	0. 00%	0. 00%	99. 68%	0. 01%
11	DP4+ (H data)		-	-	-	-	-	-	-	-
12	DP4+ (C data)		0. 00%	23. 61%	0. 00%	0. 00%	0. 00%	0. 00%	67. 24%	9. 15%
13	DP4+ (all data)		0. 00%	23. 61%	0. 00%	0. 00%	0. 00%	0. 00%	67. 24%	9. 15%

S3.2. Computational details for compound 2 (ECD)

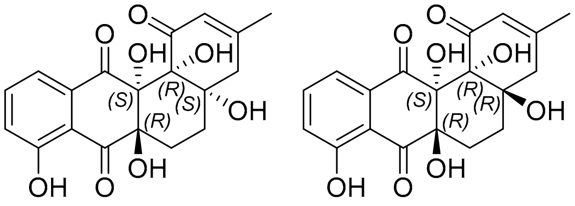

Conformation search based on molecular mechanics with MMFF force fields (Spartan'14, Wavefunction, Inc.) were performed for **2b** and **2g** gave 3 and 2 stable conformers within 20 kJ/mol, respectively. All these conformers were further optimized by the density functional theory method at the B3LYP/6-31G(d) level by Gaussian 16 program package. The ECD of stable conformers with populations higher than 1% were calculated using density functional theory (TDDFT) at B3LYP/6-31+G(d) level in methanol with IEFPCM model. The calculated ECD curves were all generated using SpecDis 1.71 with $\sigma = 0.22$ eV, and UV shift -25 and +5 nm, respectively.

Figure S6. Comparison of the calculated ECD spectra for **2b** and **2g** with the experimental spectrum of **2** in methanol with PCM model.

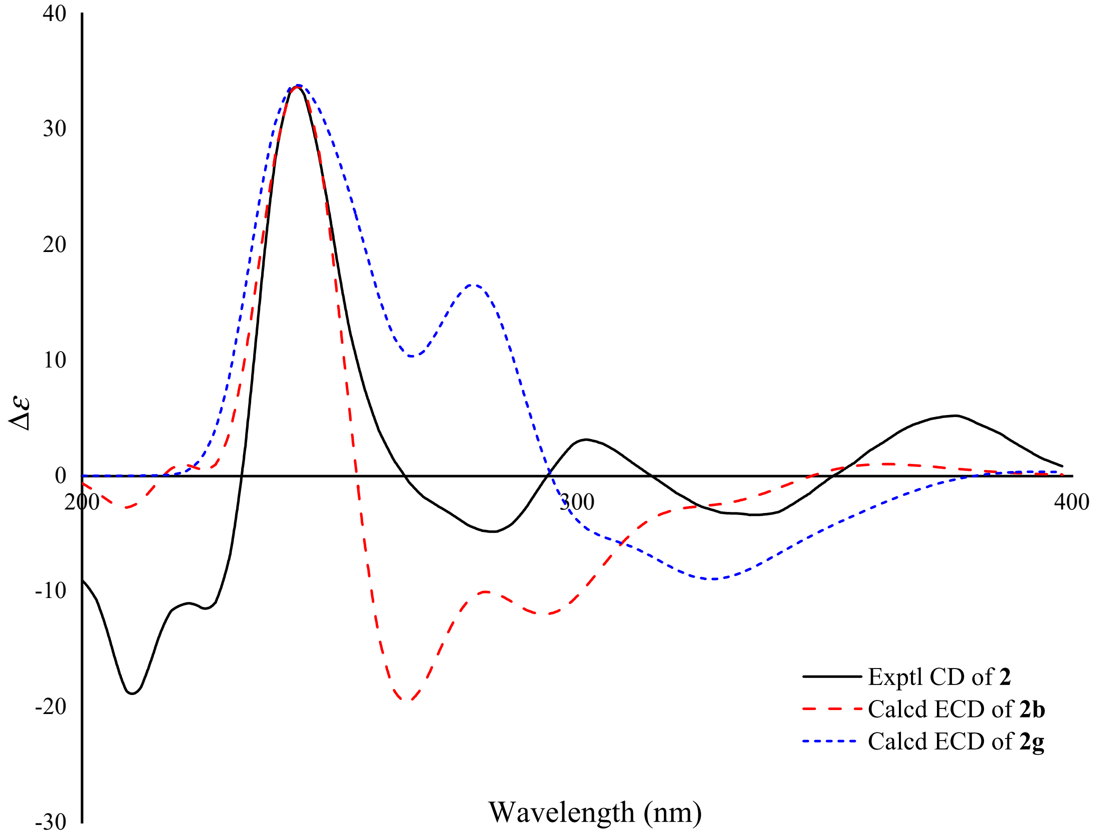

Section S4. NMR, HRESIMS, and IR spectra of 1

Figure S7. ^{1}H NMR of **1** in DMSO-d_6

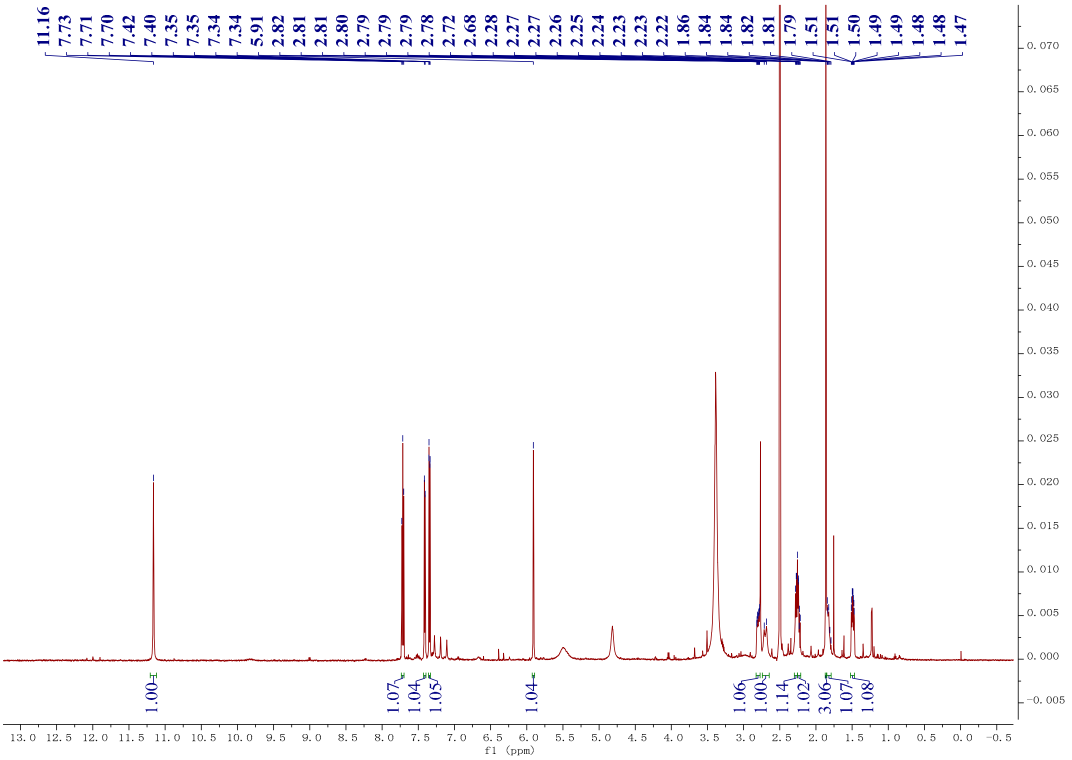

Figure S8. ^{13}C NMR of **1** in DMSO-d_6

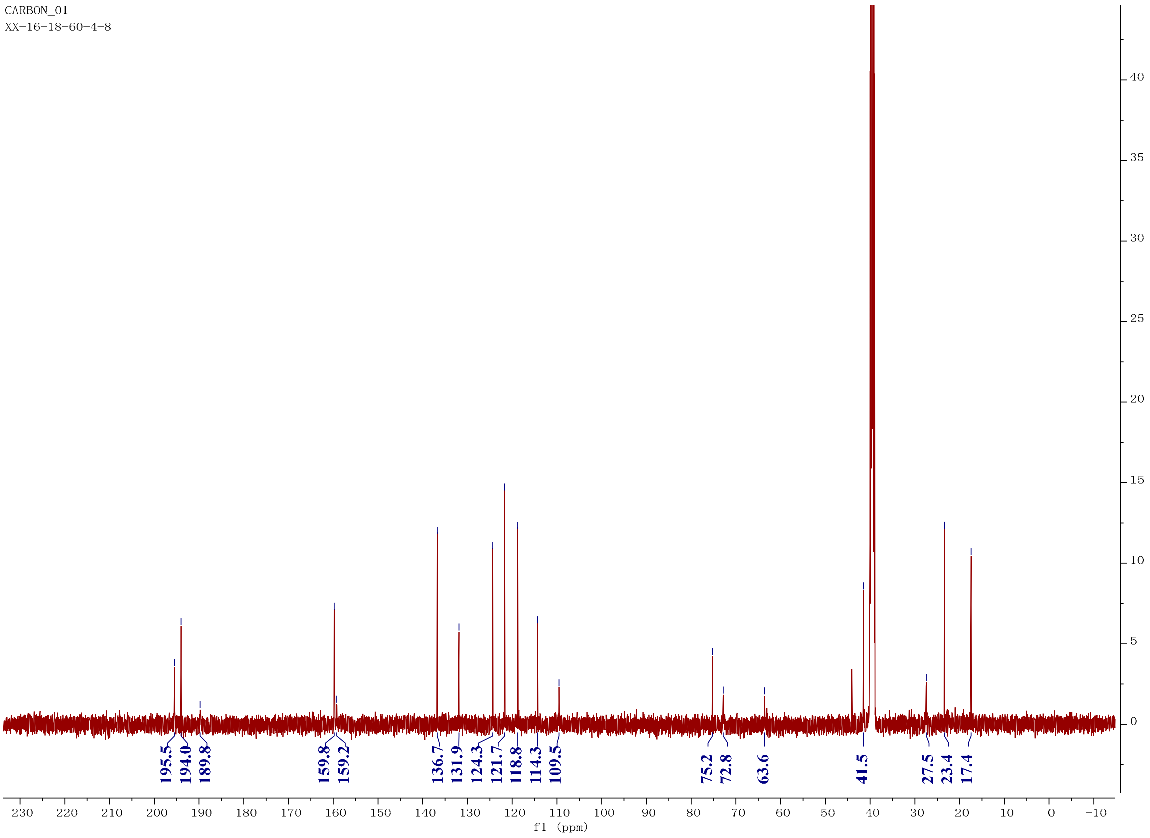

Figure S9. HSQC of **1** in DMSO-d_6

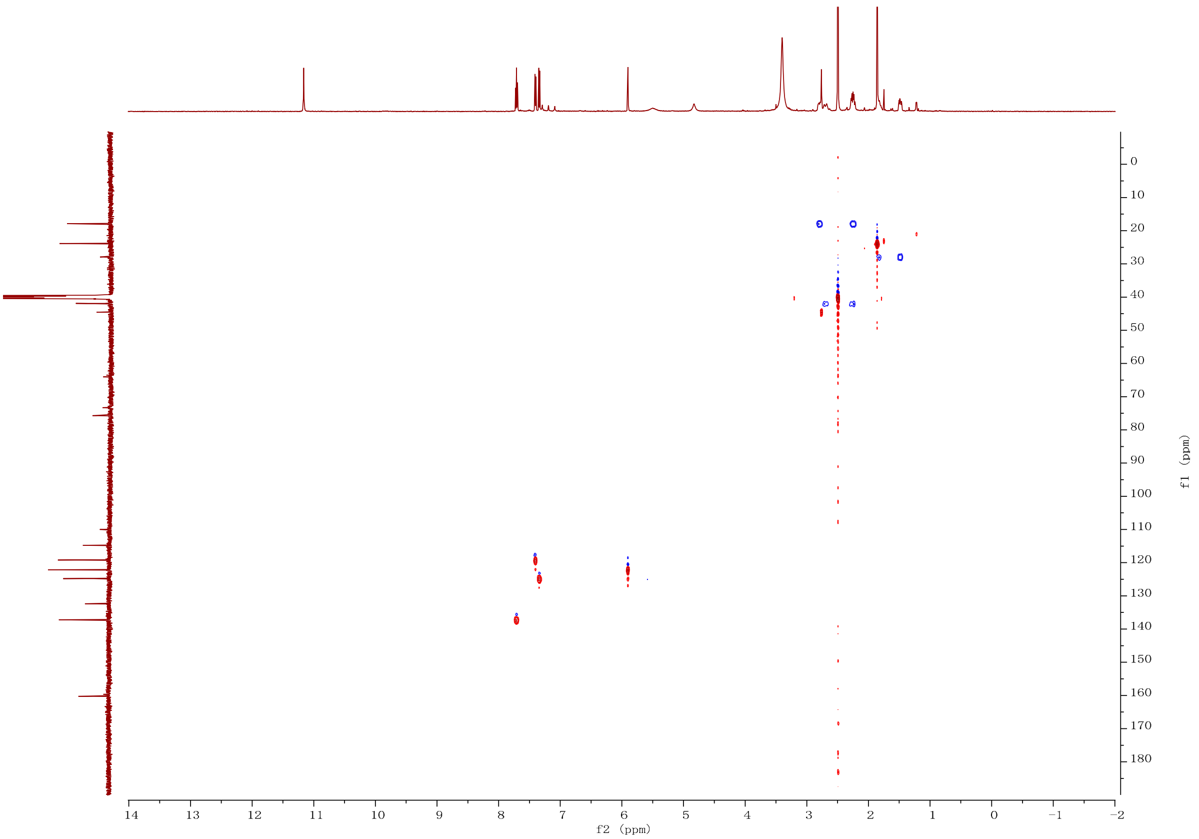

Figure S10. HMBC of **1** in DMSO-d_6

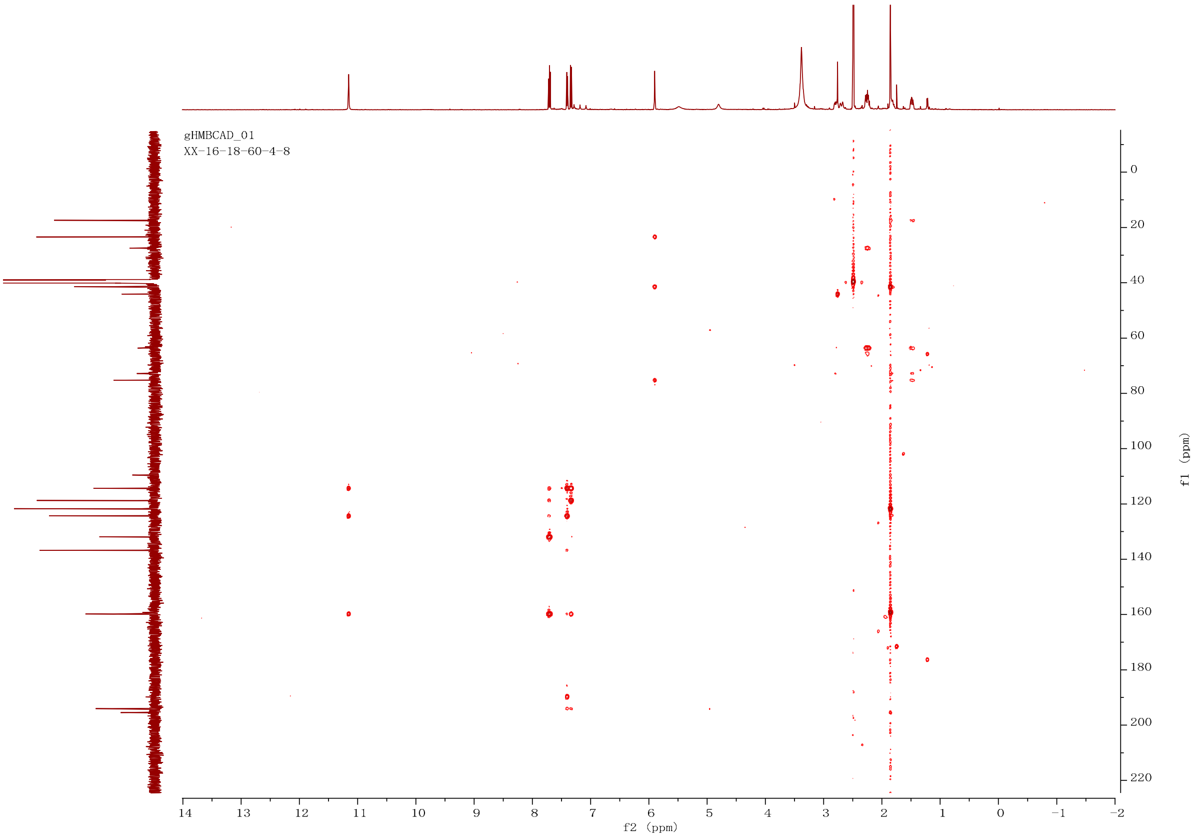

Figure S11. ^{1}H-^{1}H COSY of **1** in DMSO-d_6

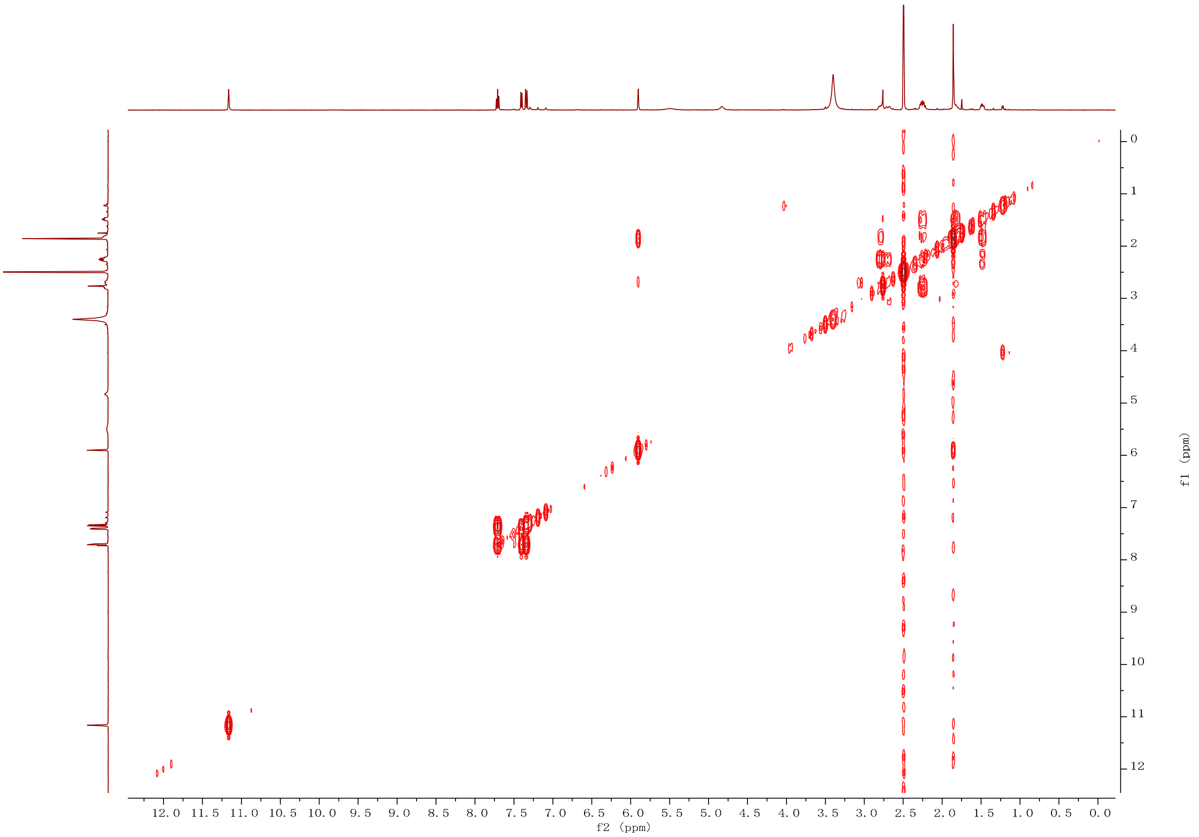

Figure S12. HRESIMS of **1**

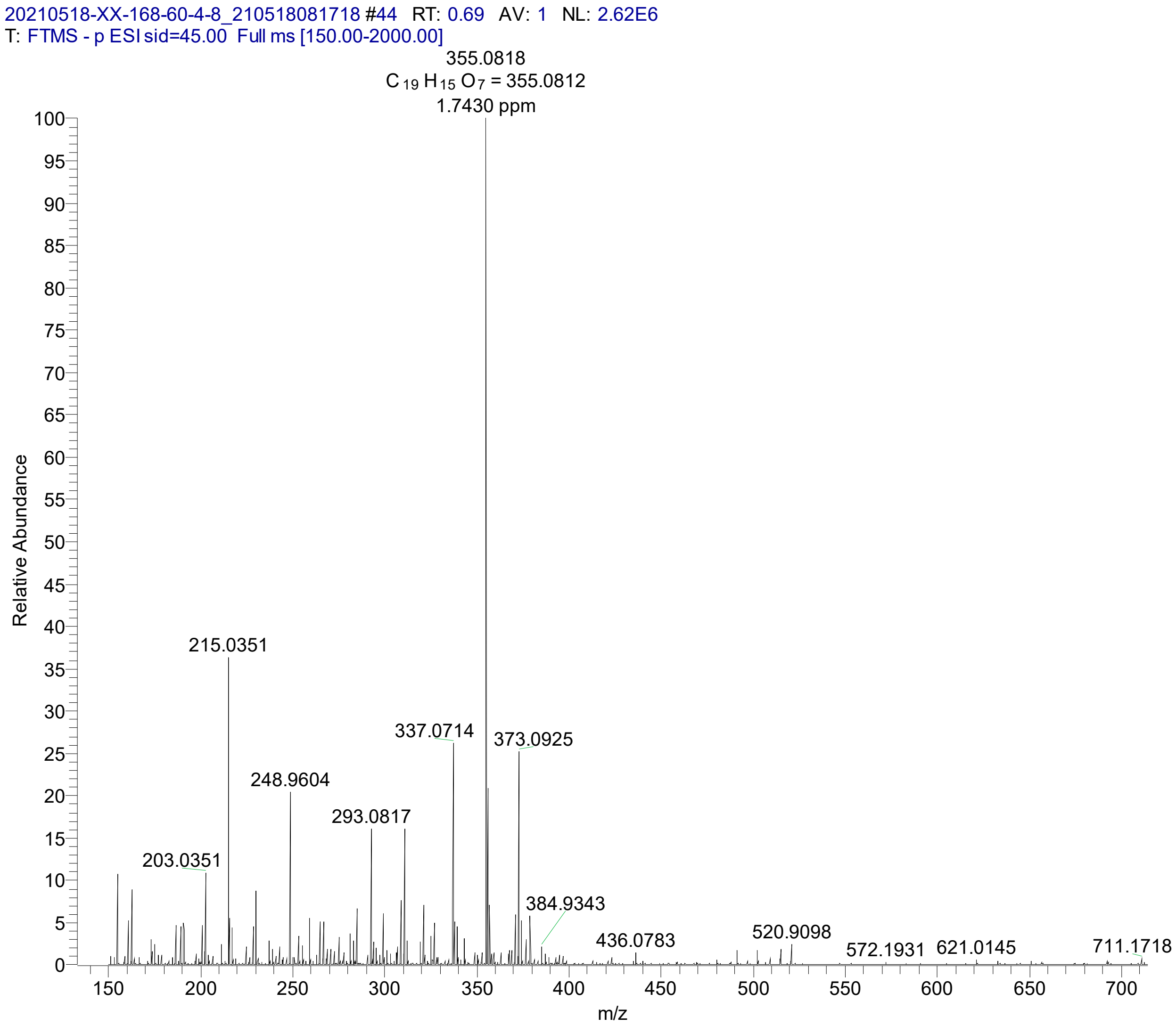

Figure S13. IR of **1**

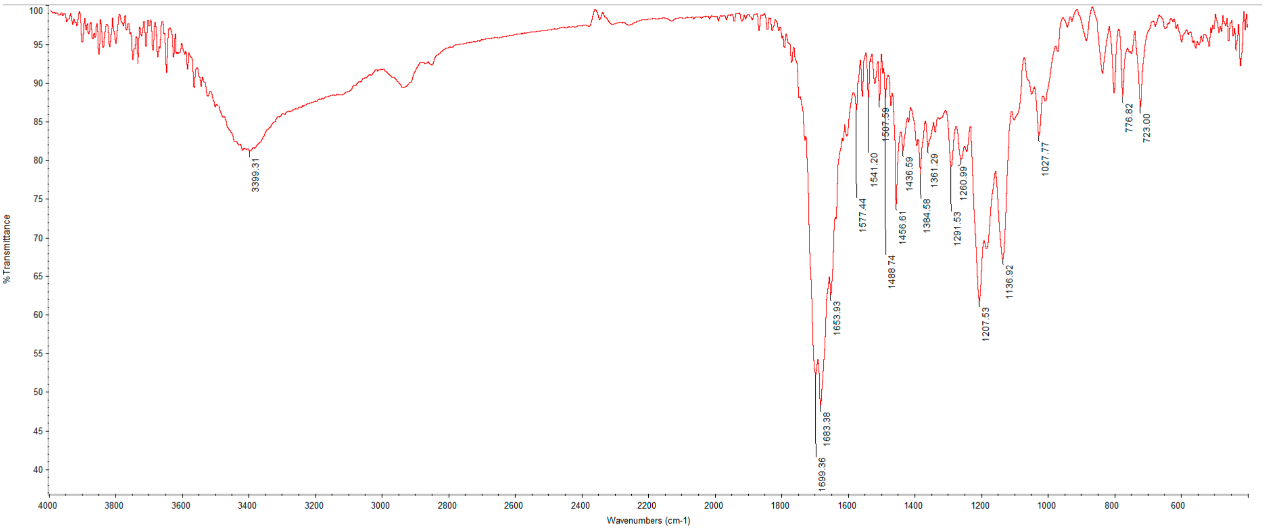

Section S5. NMR, HRESIMS, and IR spectra of 2

Figure S14. ^{1}H NMR of **2** in DMSO-d_6

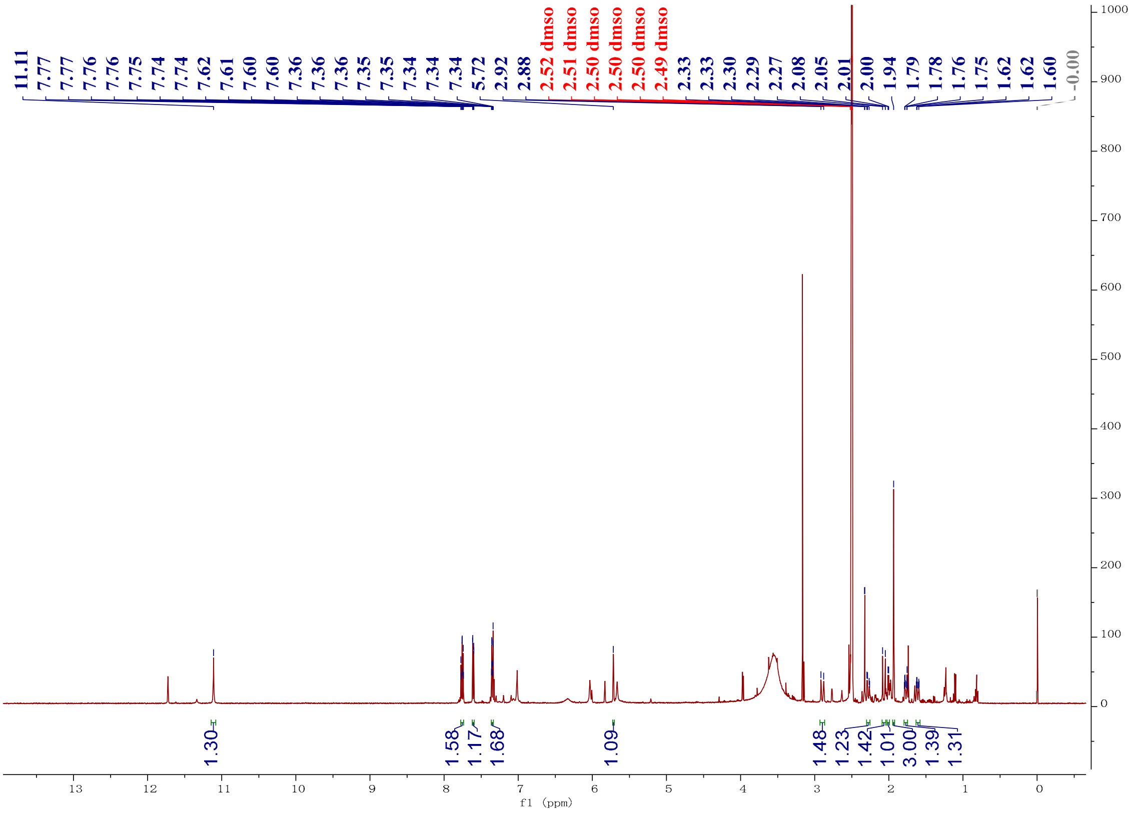

Figure S15. ^{13}C NMR of **2** in DMSO-d_6

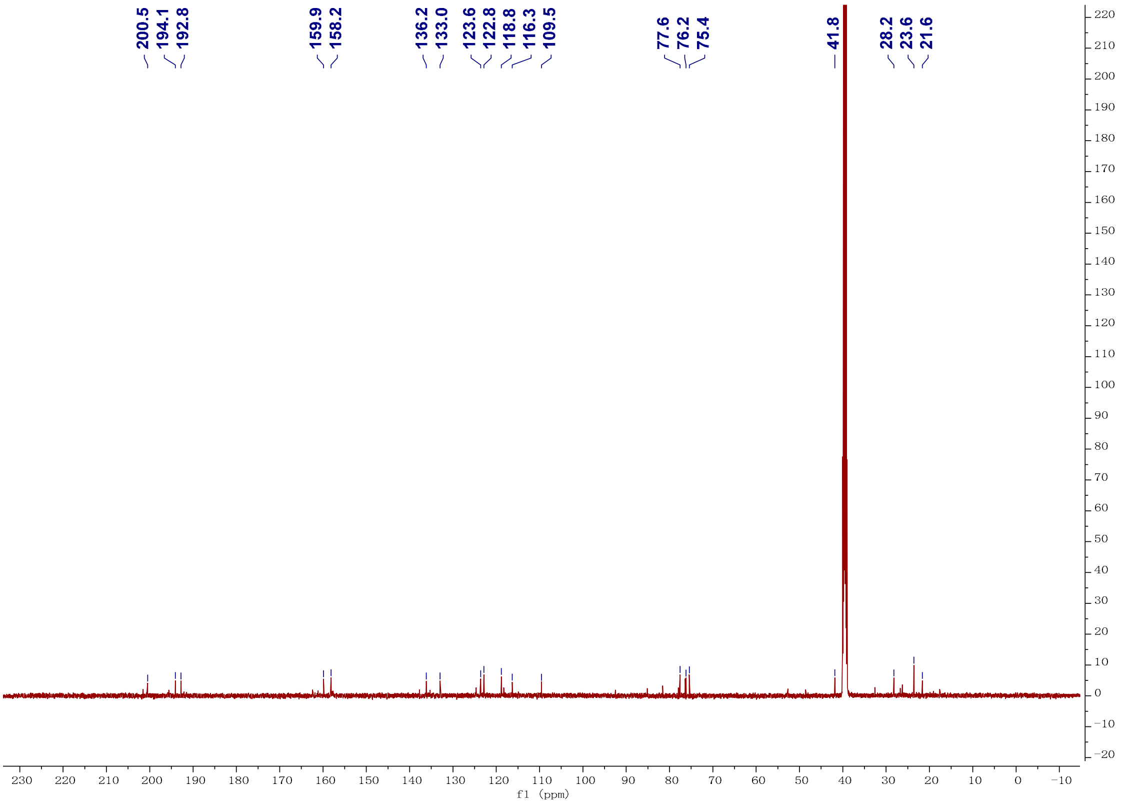

Figure S16. HSQC of **2** in DMSO-*d*6

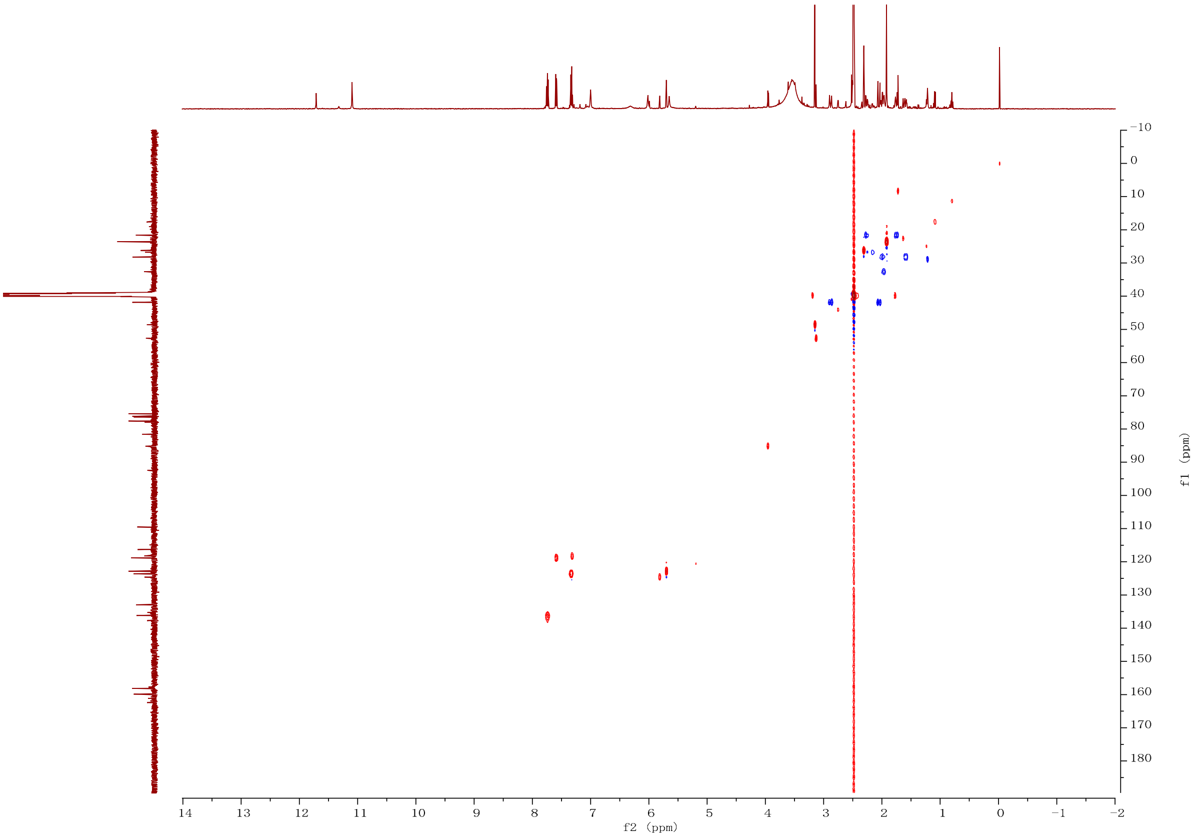

Figure S17. HMBC of **2** in DMSO-*d*6

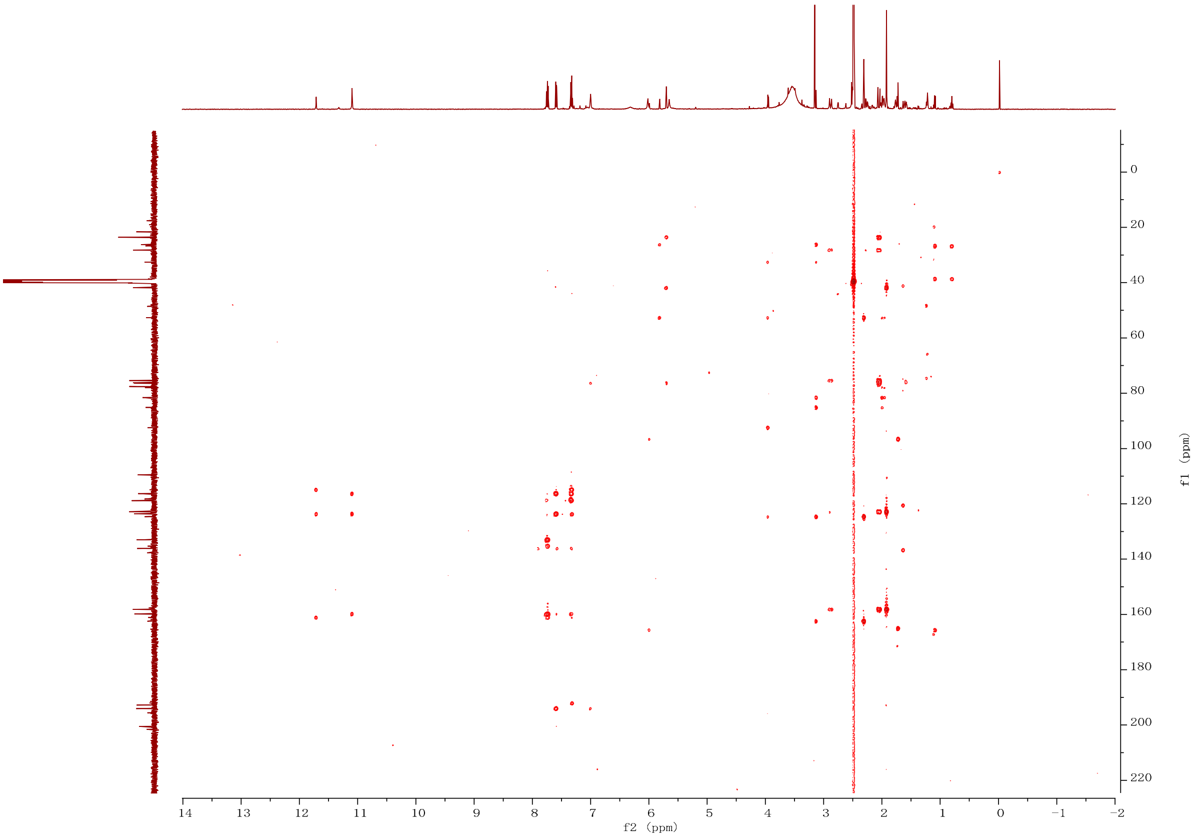

Figure S18. ^{1}H-^{1}H COSY of **2** in DMSO-d_6

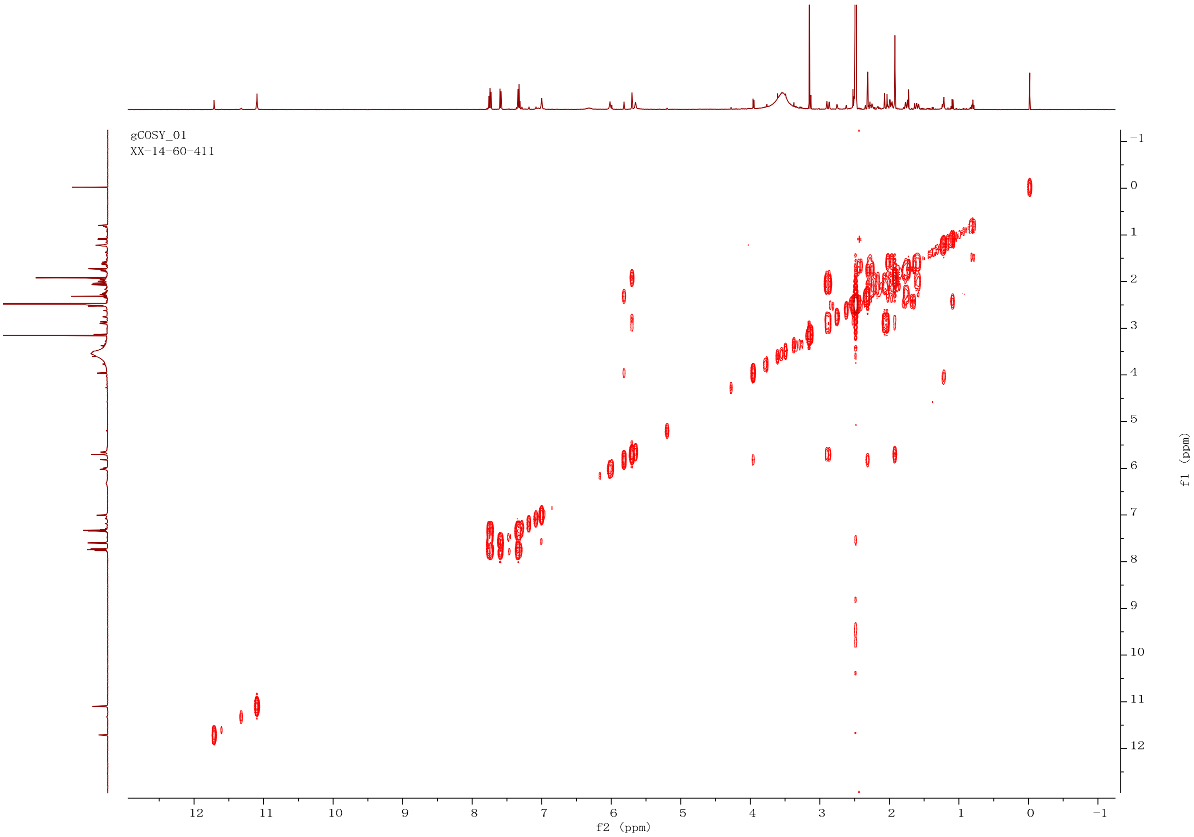

Figure S19. HRESIMS of **2**

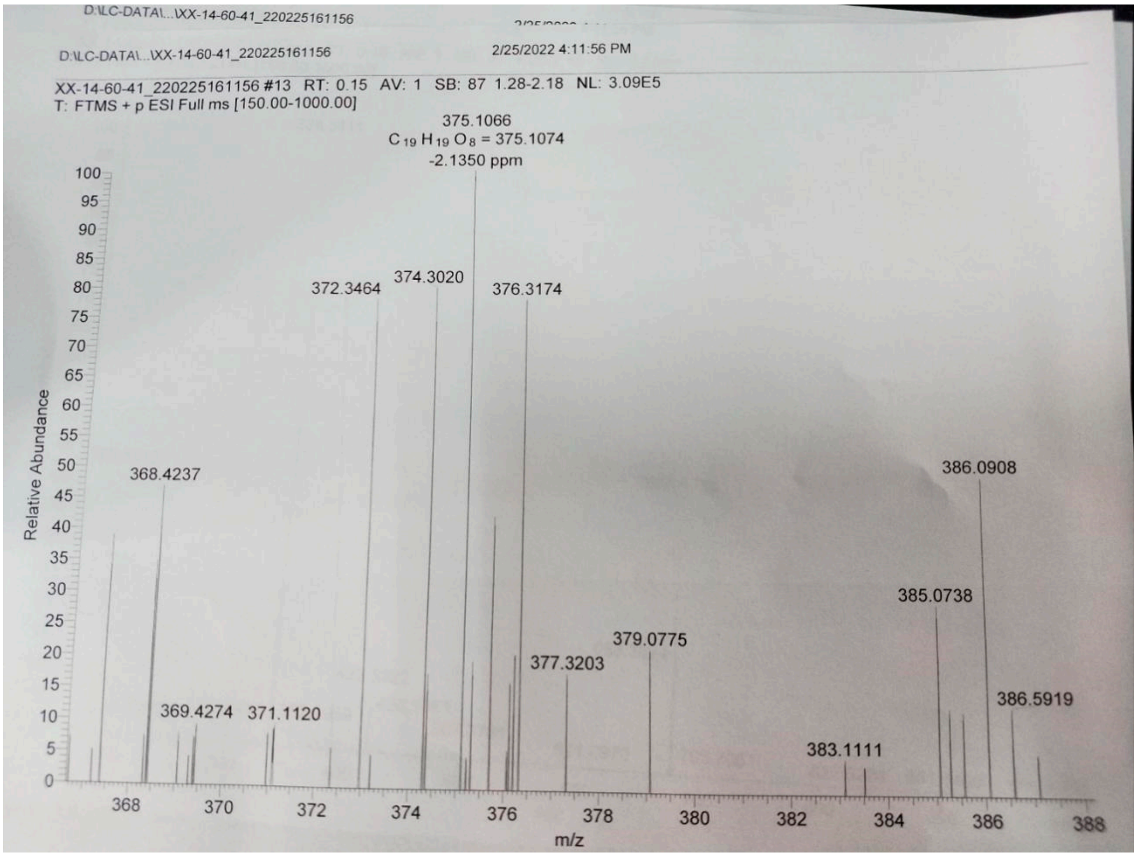

Figure S20. IR of **2**

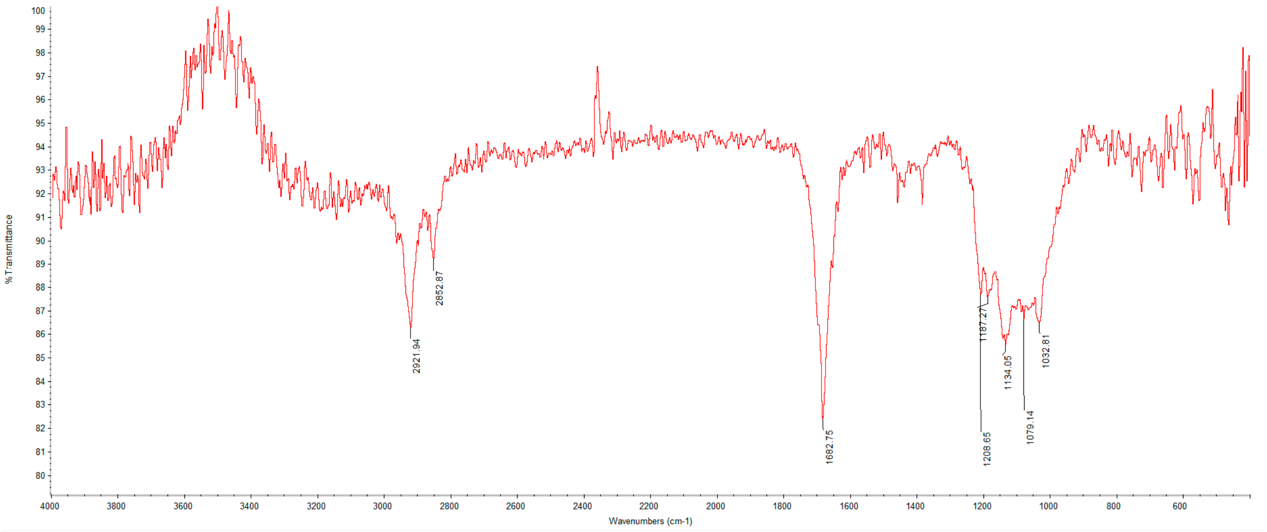

Section S6. NMR, HRESIMS, and IR spectra of 3

Figure S21. ^{1}H NMR of **3** in DMSO-d_6

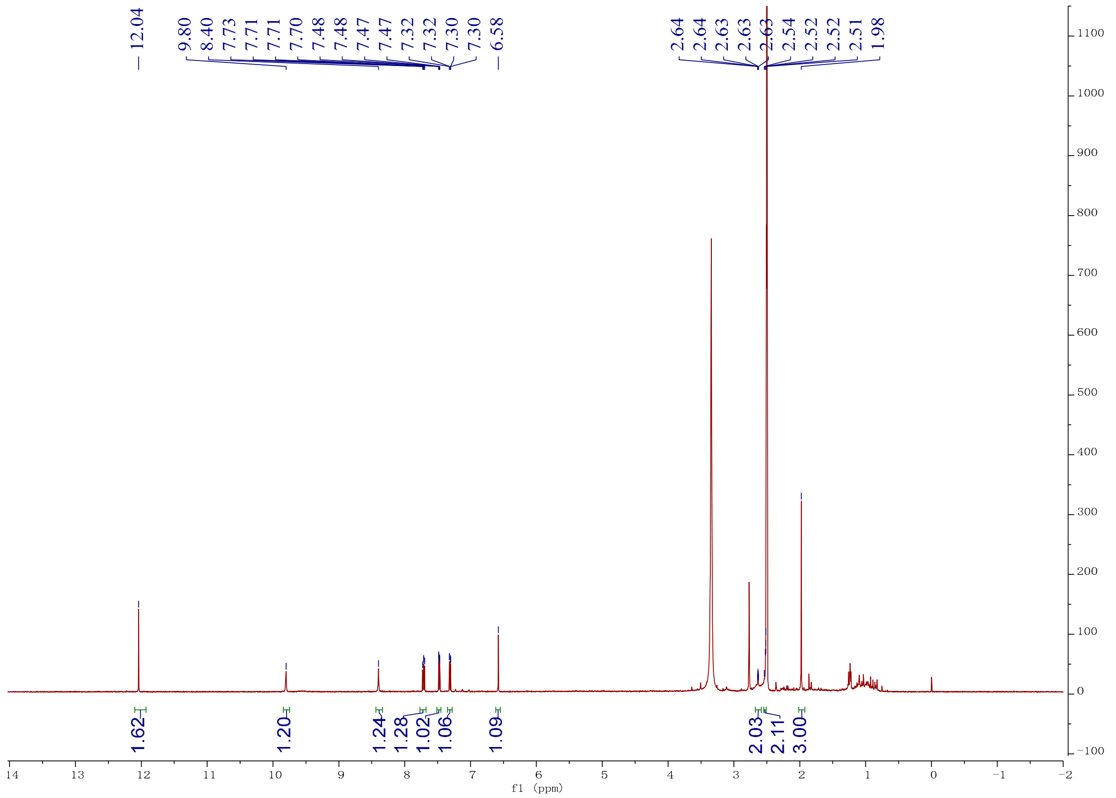

Figure S22. ^{13}C NMR of **3** in DMSO-d_6

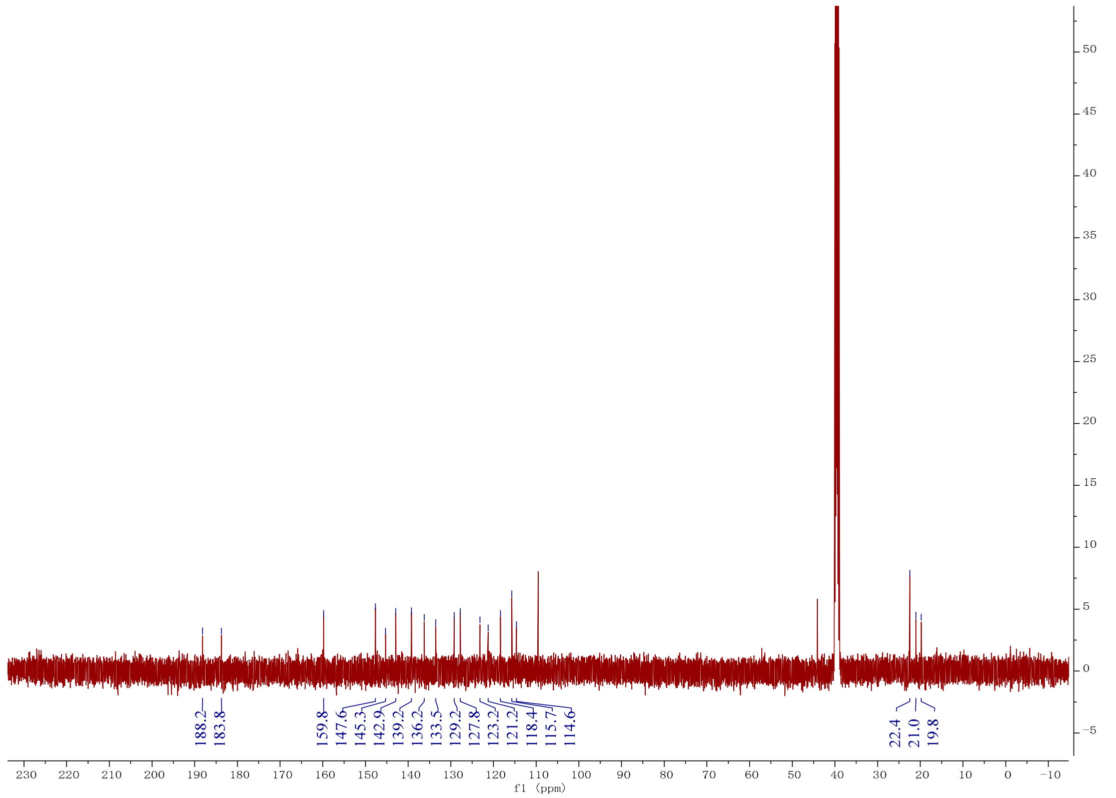

Figure S23. HSQC of **3** in DMSO-d_6

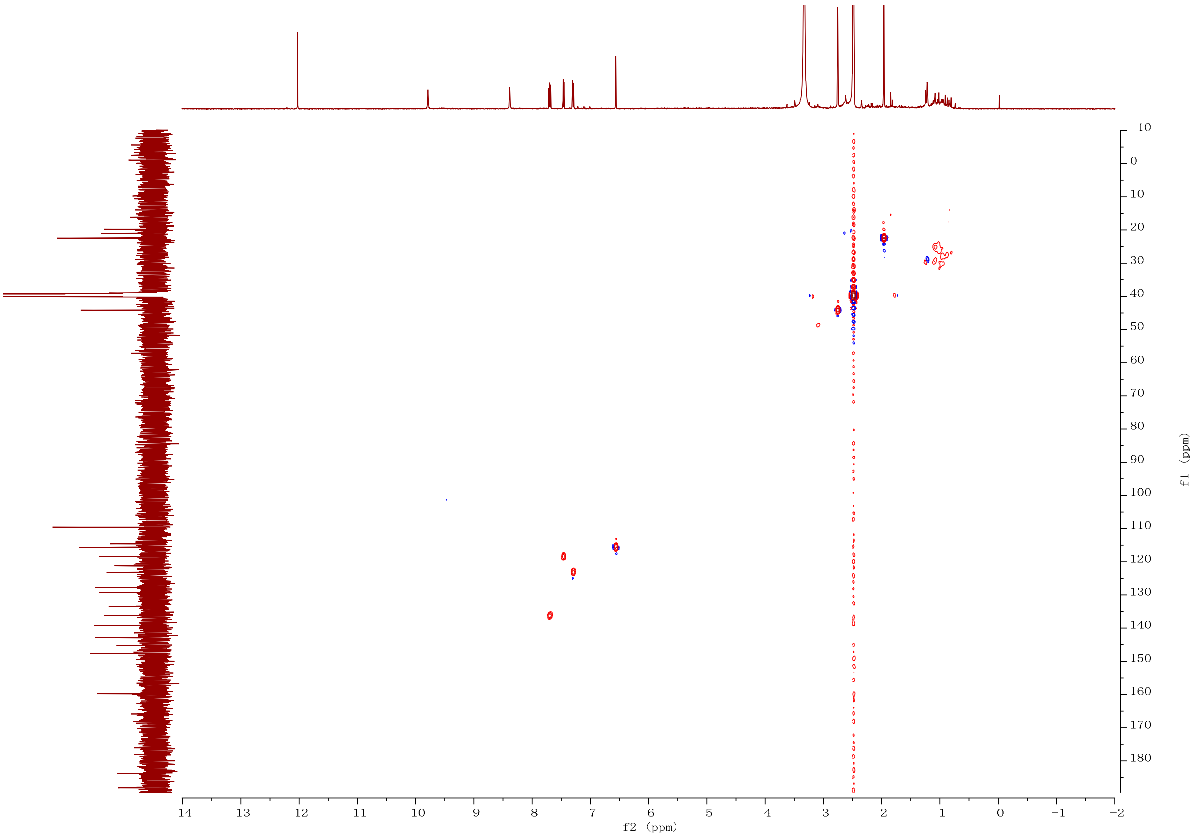

Figure S24. HMBC of **3** in DMSO-d_6

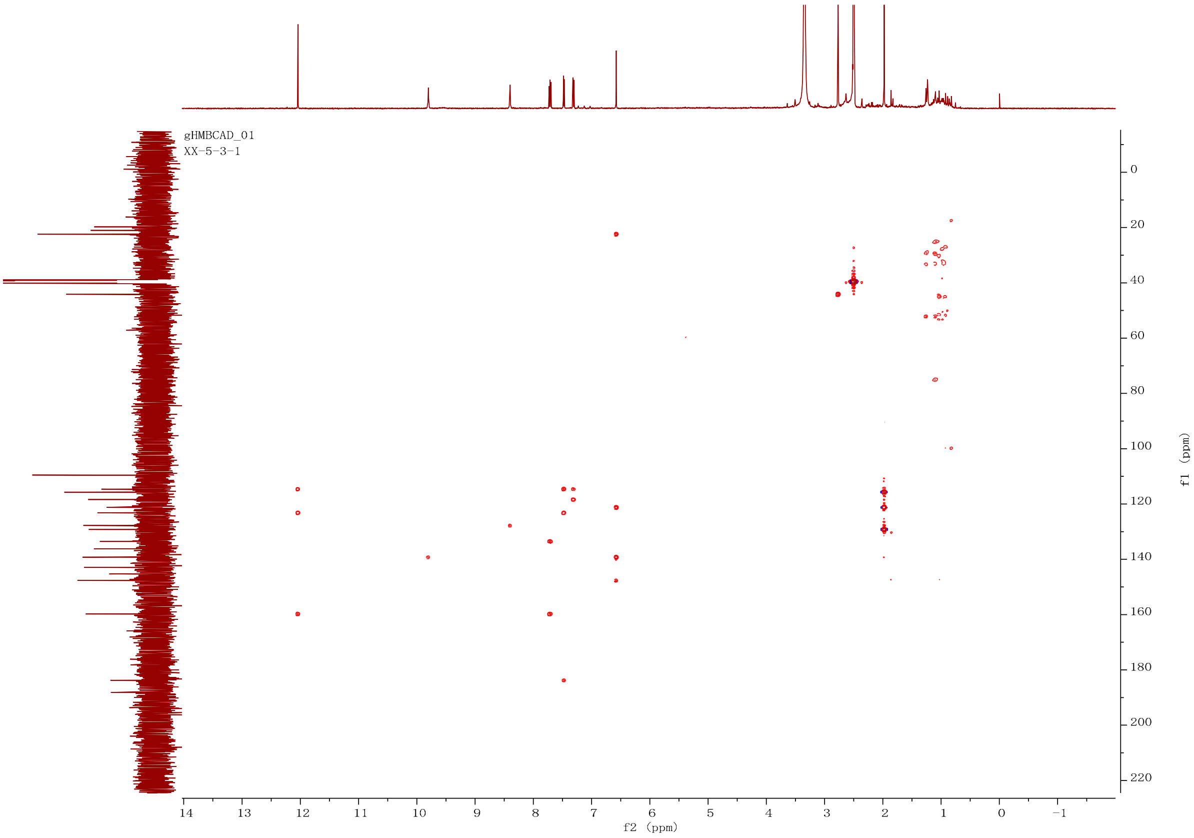

Figure S25. ^{1}H-^{1}H COSY of **3** in DMSO-d_6

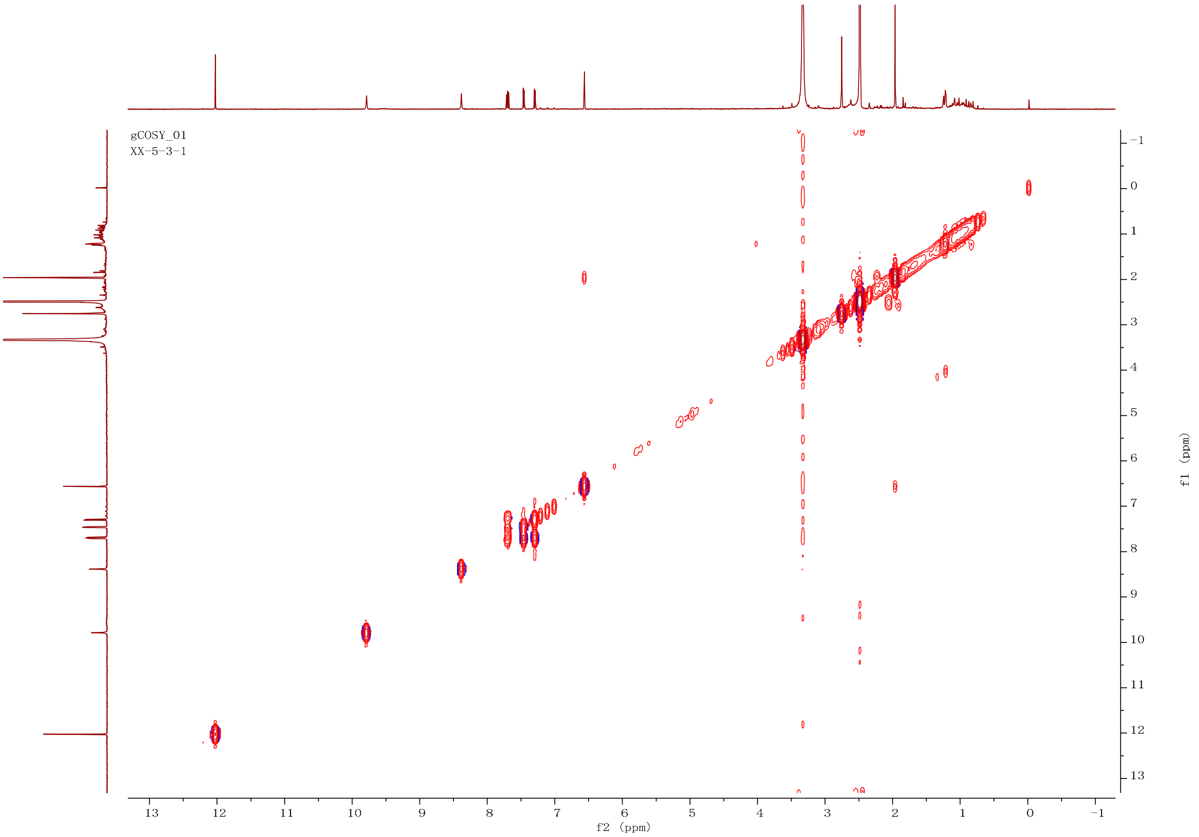

Figure S26. NOESY of **3** in DMSO-d_6

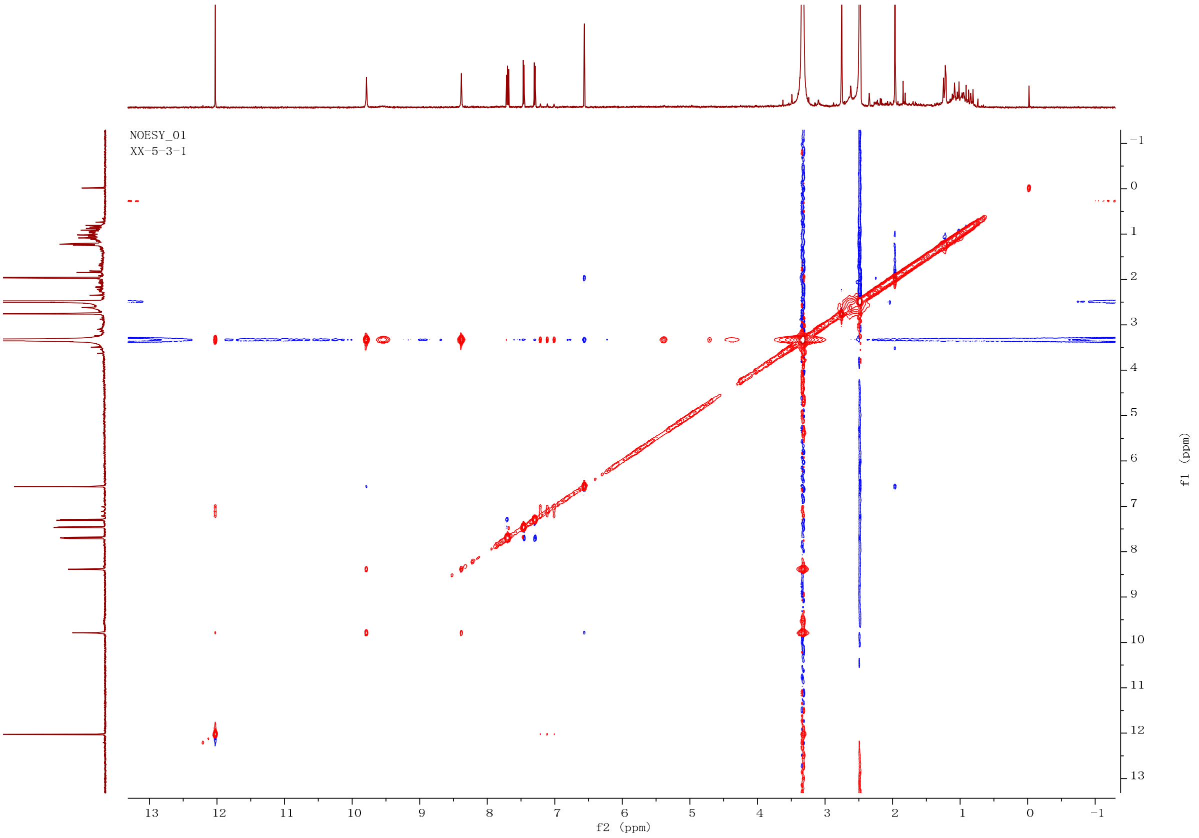

Figure S27. HRESIMS of **3**

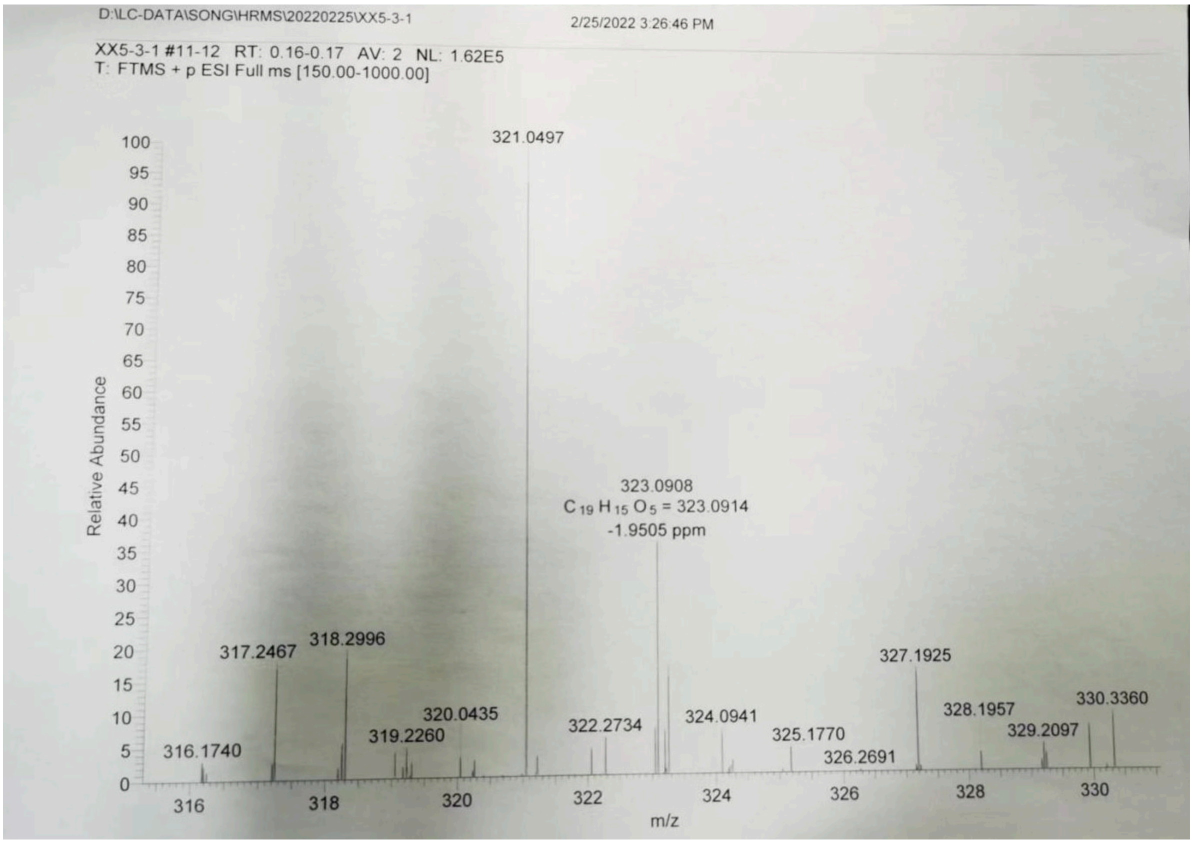

Figure S28. IR of **3**

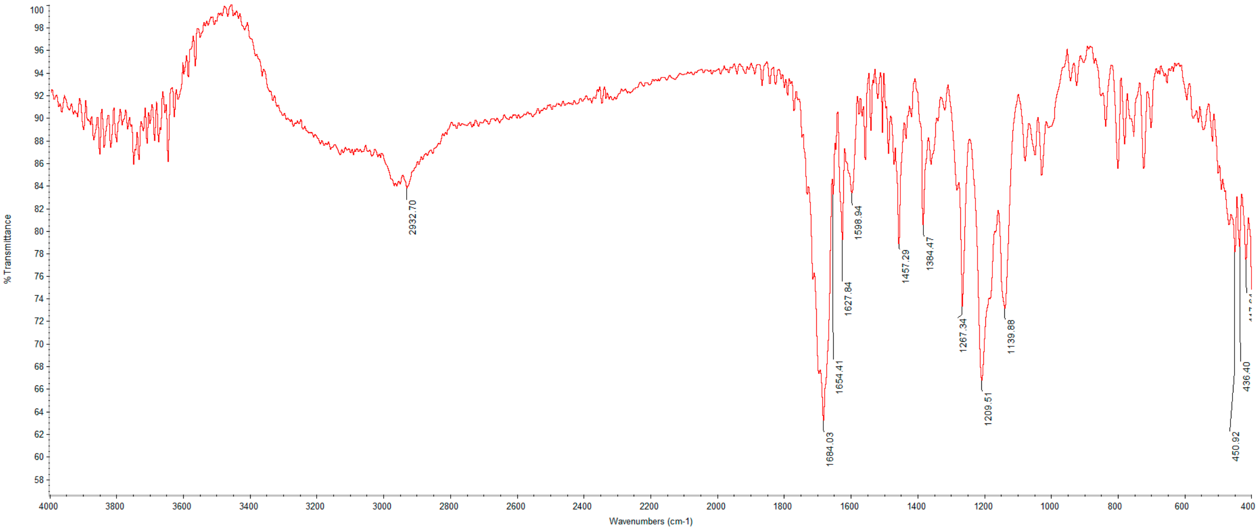

Supplement: Supplementary file 1 [file marinedrugs-21-00240-s001.zip › marinedrugs-2266681-supplementary.pdf]
